# Enhancing nurse competence in early recognition of cardiotoxicity

**DOI:** 10.1186/s40959-024-00261-x

**Published:** 2024-09-14

**Authors:** Jeff Kolbus, Mopelola T. Adeola, Janelle M. Tipton, Caitlin E. D. Luebcke

**Affiliations:** 1 Department of Nursing, Purdue University, 502 N University St, West Lafayette, IN 47907 USA; 2 Indiana Internal Medicine Consultants, 701 E County Line Rd # 101, Greenwood, Indianapolis IN 46143 USA

**Keywords:** Cardio-oncology, Cardiotoxicity, Competence, Education, Nursing, Self-efficacy

## Abstract

**Background:**

Preliminary research reveals that many nurses feel inadequate and possess limited knowledge when it comes to managing cardiotoxicity, underscoring the necessity for educational programs to enhance nursing skills in this area.

**Methods:**

The aim of the study was to assess the impact of an educational intervention on nurses perceived self-efficacy in recognizing patients exhibiting symptoms of cancer treatment-related cardiotoxicity. The study was set in a 16-bed cardiac critical care unit (CCU) within a 462-bed hospital. The sample group was comprised of registered nurses (RNs) working on or floating to the CCU. The study used a within-subjects design. Participants completed a pre-education survey, attended one of six 30-minute education interventions, and completed a post-education survey. The outcome variables were 7 self-confidence questions from the Nursing Self-Efficacy Scale for Managing Cancer Treatment-Related Cardiotoxicity (NSS-CTC) on a 5-point Likert scale and one yes or no self-efficacy question. Descriptive statistics and paired T-tests were applied to analyze pre- and post-education surveys.

**Results:**

The pre-and post-education comparative analysis for each of the 7 NSS-CTC self-confidence questions was statistically significant with test statistics ranging from *t* = 3.43 to *t* = 8.69 and *p*-values ranging from 0.0021 to less than 0.0001. All 26 RNs answered “yes” in their ability to detect symptoms of cancer therapy-related cardiotoxicity after the education.

**Conclusions:**

The lack of education for cardiac nurses against the backdrop of increasing cardiotoxicity in cancer patients showcases the essential need for cardiac nurse early symptom recognition education.

**Supplementary Information:**

The online version contains supplementary material available at 10.1186/s40959-024-00261-x.

## Background

Advances in cancer treatment have improved the long-term survival of patients but have also led to life-threatening cardiac side effects. The escalating incidence of cardiovascular toxicity in cancer patients, both during and after cancer treatment, has emerged as a formidable challenge in healthcare [9]. This phenomenon, known as cardiotoxicity, manifests as detrimental effects on the heart’s structure and function due to cancer therapies. Cardiotoxicity has alarmingly ascended to become the second predominant cause of long-term morbidity and mortality among cancer treatment recipients [8, 10]. In response to this critical issue, the field of cardio-oncology has been established, aiming to refine the management of cardiovascular complications stemming from cancer treatments [7].

Nurses occupy a pivotal role in the early detection, monitoring and management of cardiotoxicity [12]. However, a concerning gap in the preparedness and competence of nursing professionals in this realm exists. Self-efficacy, a critical component linked to nursing competency, influences nursing performance, motivation, and learning outcomes [2, 4, 5, 11, 14]. Preliminary research indicates a prevalent sense of inadequacy and limited knowledge among nurses in managing cardiotoxicity [3, 8, 12]. Educational interventions are crucial to enhance nursing competence in managing cardiotoxicity, addressing the significant shortfall in knowledge and preparedness among nurses.

## Methods

The aim of the study was to assess the impact of an educational intervention on nurses perceived self-efficacy in recognizing patients exhibiting symptoms of cancer treatment-related cardiotoxicity. The objective was to elevate the competence of cardiac nurses in the early recognition of cancer therapy-related cardiotoxicity symptoms, thereby enhancing the quality of patient care and outcomes in this specialized area.

The researchers gathered data through pre- and post-education self-efficacy surveys. The study focused on nurses ability to recognize early symptoms of cancer-therapy related cardiotoxicity. The outcome variables were the 7 self-confidence questions from the Magon et al. [12] Nursing Self-Efficacy Scale for Managing Cancer Treatment-Induced Cardiotoxicity (NSS-CTC) on a 5-point Likert scale that ranged from no confidence = 1 to high confidence = 5 and one yes or no self-efficacy question.

The NSS-CTC (Appendices A, B, and C) is a comprehensive 15-item self-report tool created to evaluate nursing self-efficacy in addressing cancer treatment-related cardiotoxicity. This scale encompassed a balanced mix of five knowledge-centric questions and ten practice- oriented questions. Eight questions (5–13, and 15) were excluded from the survey since they were less pertinent to cardiac nurses roles in recognizing and monitoring cardiotoxicity symptoms, particularly for non-oncology nurses. The NSS-CTC has demonstrated psychometric reliability, underpinned by content assessment of knowledge-related self-efficacy (Cronbach’s α = 0.924) and practice-related self-efficacy (Cronbach’s α = 0.937) [12]. Utilization of the NSS-CTC in this study is sanctioned by the authors. Additionally, a range of demographic variables were captured of survey participants profiles, ensuring comprehensive data analysis.

### Study design and procedure

The study used a within-subjects design and incorporated baseline data collection, intervention implementation, and post-intervention data collection. Participants completed a pre-education survey, attended one of six 30-minute education interventions, and completed a post-education survey. The 30-minute educational intervention was delivered on six separate occasions to accommodate all participants, ensuring each nurse attended one session. The educational intervention aimed to enhance nurses’ competence in early recognition of cardiotoxicity. The educational interventions were conducted on January 3rd, 4th, and 5th. Sessions for the night shift nurses began at 6:00 AM, while those for the day shift started at 8:00 AM. This study received hospital-site IRB approval on November 20, 2023, and IRB approval from Purdue University on December 18, 2023.

### Educational intervention description

The educational component of the study was a structured intervention titled “Improving Nurse Early Cancer Related Cardiotoxicity Symptoms Recognition,” which aimed to enhance nurses’ capabilities in identifying early signs of cardiotoxicity in cancer patients. This intervention, delivered in a single 30-minute session, was repeated across six separate occasions to accommodate varying nurse schedules. The session comprehensively covered the following key areas:

1. **Cardiotoxicity and Cardio-oncology Overview**: Introduced the concept of cancer-therapy related cardiotoxicity and the fundamentals of cardio-oncology, emphasizing the importance of early detection and intervention.
2. **Identification of Cardiotoxic Medications**: Detailed the specific chemotherapeutic agents known for their cardiotoxic effects, teaching nurses how to recognize symptoms and understand the underlying mechanisms of action leading to cardiovascular complications.
3. **Cardio-oncology Care Practices**: Explained the use of the Cardio Oncology Surveillance Tool within the Epic Flowsheets, focusing on how to document medication-related risks and assess patient-related risk factors effectively.
4. **Patient Education and Advocacy**: Emphasized the role of nurses in educating patients and their families about the risks of cardiotoxicity and the critical need for ongoing monitoring and management.

Interactive teaching methods, including case studies and real-life scenarios, were employed to engage participants and enhance the practical application of the content. Each nurse also received a reference manual to reinforce the educational material and provide a resource for ongoing reference. This intervention was designed to directly translate into improved patient care practices, equipping nurses with the necessary knowledge and skills to address the complex needs of cancer patients at risk of cardiotoxic events.

### Sample and setting

In this study, a total of 45 RNs working in the CCU were eligible to participate. Of these, 30 nurses were initially targeted for the study based on their availability and willingness to participate during the intervention dates. Ultimately, 26 nurses completed both the pre- and post-intervention.

The study was set in a CCU of a major Midwest city hospital, targeting cardiac nurses caring for an oncology patient population at risk for cardiotoxicity. The unit, a 16-bed CCU within a 462-bed hospital, presented an ideal environment for this research.

### Sample and setting relevance

This setting is particularly relevant as it frequently admits patients who are at risk for cardiotoxicity, especially those undergoing cancer treatments known to have cardiotoxic effects. All CCU nurses who care for patients receiving cancer treatment must be familiar with potentially cardiotoxic cancer medications and treatments, recognize the signs and symptoms of cardiotoxicity, and know when to refer patients to cardio-oncology for further assessment and treatment. The CCU nurses play a crucial role in recognizing symptoms and communicating these findings to cardiologists, many of whom also serve as cardio-oncology providers.

**Fig. 1:**
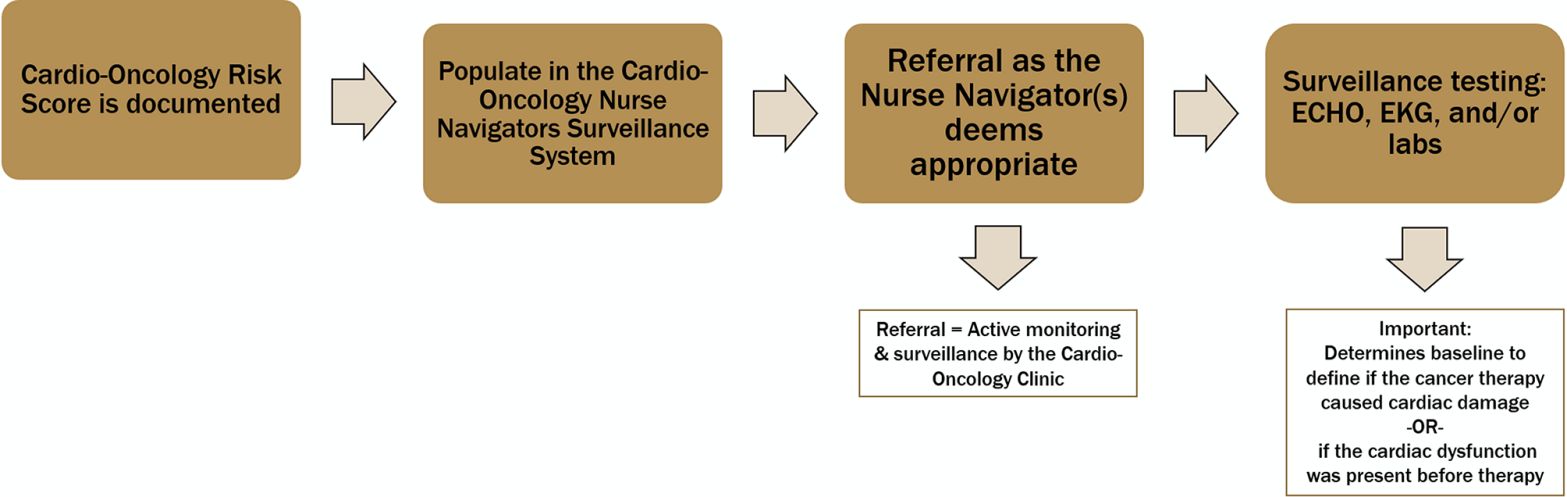
Illustrates the Cardio-Oncology Risk Score Electronic Health Record charting in the Epic system

**Fig. 2:**
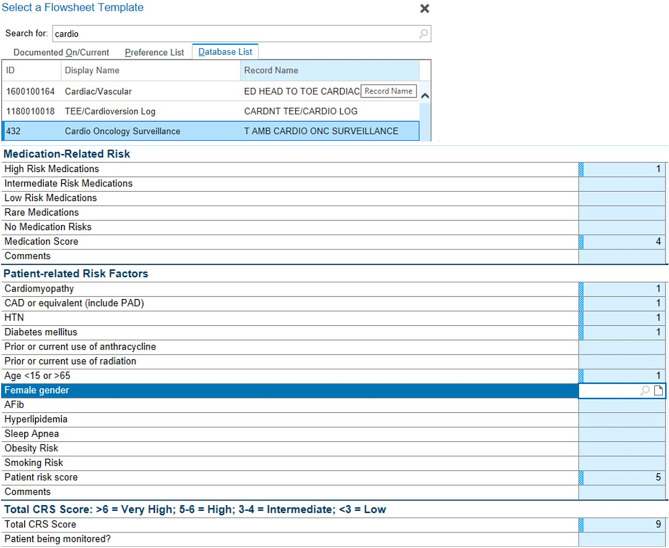
Shows the interface of the Cardio Oncology Surveillance Tool used within the hospital

The hospital utilizes the Cardio-Oncology Risk Score (CRS) within the Cardio Oncology Surveillance Tool integrated into the Epic electronic health record (EHR) system (Figs. 1 and 2). This tool facilitates a two-way referral system between cardiology and oncology. When the CRS indicates an intermediate, high, or very high risk, the cardio-oncology nurse navigator receives an automatic notification, prompting an immediate referral. The nurse navigator then determines if the patient meets the criteria for surveillance monitoring.

**Fig. 3:**
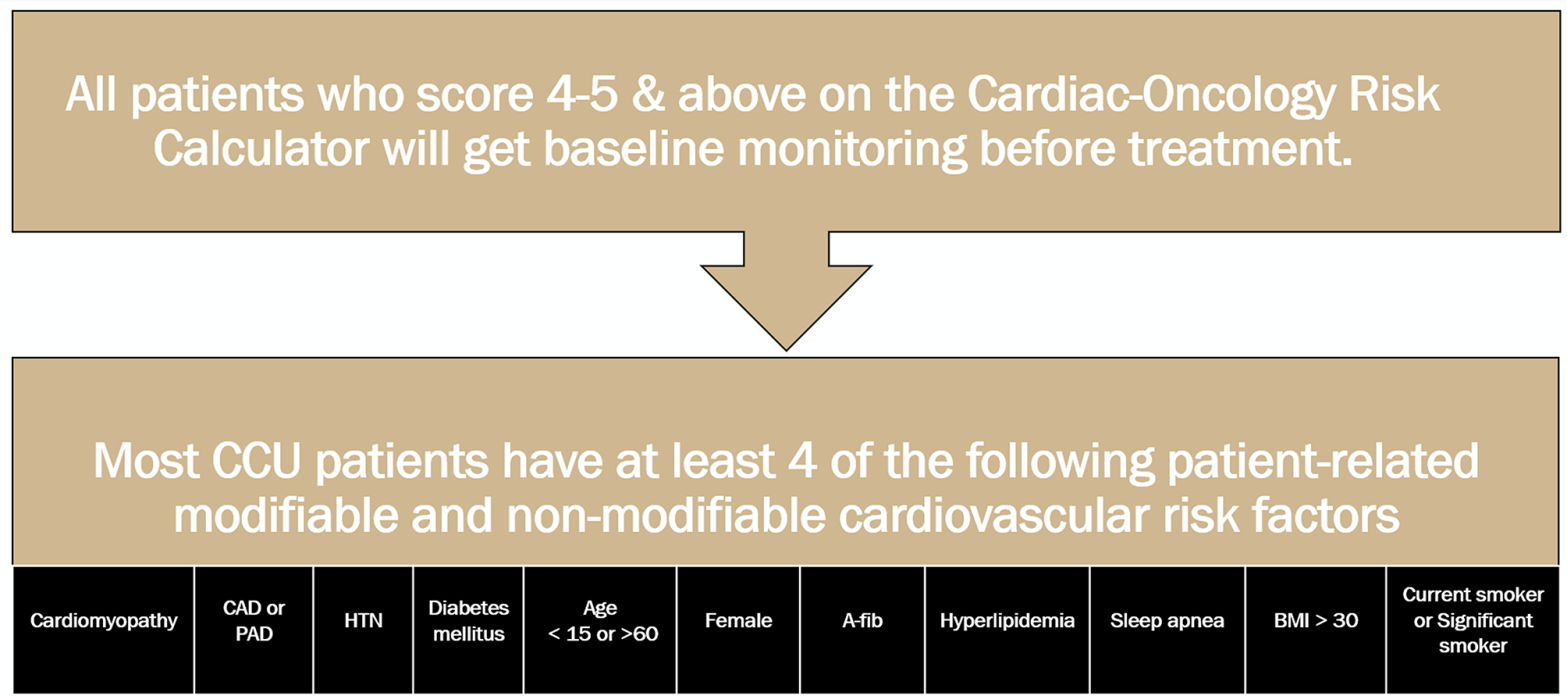
Depicts the distribution of high cardiac risk factors among the CCU patient population, emphasizing the critical need for specialized nursing skills in this area

Given the high cardiac risk factors prevalent in the CCU patient population (Fig. 3), the setting provides an ideal environment for studying the impact of educational interventions aimed at enhancing nurse competence in recognizing and managing cardiotoxicity. The utilization of the CRS and the Cardio Oncology Surveillance Tool ensures that RNs in the CCU can effectively place patients into appropriate cardio-oncology care pathways, thereby improving patient outcomes through timely and accurate assessments. This strategic use of technology and team-based care not only underscores the relevance of the CCU as a critical study site but also highlights the importance of nurse training in specific areas of cancer treatment-related cardiotoxicity complications.

This detailed setup within the CCU provides a clear example of how specialized environments can significantly benefit from targeted educational programs, which are designed to enhance specific competencies that are crucial in high-stakes healthcare scenarios. The integration of these tools and protocols within the CCU ensures that the study’s findings are not only applicable but also essential for advancing nursing practices in cardio-oncology.

### Privacy and confidentiality measures

To uphold privacy and confidentiality, all pre- and post-education survey data was anonymized. A unique participant identification system, involving partial personal identifiers, was employed to link pre- and post-project data, ensuring data integrity while safeguarding participant anonymity. The university Human Research Protection Program (HRPP) and the hospital site HRPP both determined the study qualified for exempt status from International Review Board review.

### Data analysis

The alpha level chosen for statistical significance was < 0.05 while the Statistical Package for Social Sciences (SPSS) was used for data analysis. Data were collected at two time points, utilizing questionnaires derived from Qualtrics, a data collection software. Descriptive statistics were used to analyze the following: participants demographics, pre- and post-education survey mean (M), pre- and post-education survey standard deviation (SD), and the responses to a self-efficacy yes or no question. Paired T-tests were used to analyze variations in the responses to the 7 NSS-CTC self-confidence questions. The Bonferroni correction was used to control the family-wise error rate, adding rigor to the analysis. To keep the probability of at least one false positive across all 7 tests at 5%, the *p*-values for each test were compared to the multiple testing corrected cut off threshold of 0.00625 (i.e., 0.05/7).

## Results

Demographic variables assessed (Table 1) included age, gender identity, employment status, years worked as a RN, highest nursing degree completed, highest non-nursing degree completed, previous oncology nursing experience, and primary unit of employment. The number of possible participants included 45 RNs. The target population was 30 RNs. The total number of subjects who completed the surveys was 26 RNs.

**Table 1:** Demographic characteristics of participants

Demographic Variable		Sample
	N	%
Age		
20–30	12	46.2%
31–40	11	41.3%
41–50	2	7.7%
51+	1	3.8%
Gender Identity		
Female	20	76.9%
Male	6	23.1%
Transgender	0	0%
Other	0	0%
Prefer not to answer	0	0%
Employment Status		
Full-time	21	80.8%
Part-time	3	11.5%
PRN	2	7.7%
Number of Years of RN Experience		
< 1 year	3	11.5%
1–4 years	7	26.9%
5 –9 years	9	34.6%
10–14 years	7	26.9%
15–19 years	0	0%
20 + years	0	0%
Highest Nursing Degree Completed		
Associate Degree in Nursing	2	7.7%
Bachelor’s Degree in Nursing	22	84.6%
Master’s Degree in Nursing	2	7.7%
Doctorate Degree in Nursing	0	0%
Highest Nursing Non-Degree Completed		
Associate Degree (Non-Nursing)	0	0%
Bachelor’s Degree (Non-Nursing)	13	50%
Master’s Degree (Non-Nursing)	0	0%
Doctorate Degree (Non-Nursing)	0	0%
Previous Oncology Nursing Experience		
Yes	0	0%
No	26	100%
Primary Unit of Employment		
CCU	21	80.8%
Resource ICU Nurse	2	7.7%
Travel ICU Nurse	2	7.7%
Float ICU Nurse	1	3.8%

The 20–30 age group included 12 RNs (46%) and the 31–40 age group included 11 RNs (42%). Female participants included 20 RNs (76.9%). RNs employed full-time included 21 participants (80.8%). Seven participants (27%) had 1–4 years of experience as a RN and 9 (35%) had 5–9 years of experience. In the highest nursing degree completed, 22 RNs (85%) had a bachelor’s degree in nursing; in the highest non-nursing degree completed, 13 RNs (50%) had a bachelor’s degree. None of the 26 participant RNs reported having prior oncology nursing experience. The CCU was the primary unit of employment for 21 RNs (80.8%).

Table 2 summarizes the descriptive statistics and the results of the paired t-tests conducted to evaluate the educational intervention’s effectiveness. The NSS-CTC survey contained 7 self-confidence questions on a 5-point Likert scale rated 1 to 5 with 1 = no confidence, 2 = little confidence, 3 = some confidence, 4 = confidence, 5 = high confidence. Descriptive statistics and paired T-tests were applied to analyze pre- and post-education surveys from a sample of 26 RNs (N = 26), with statistical significance set at *p* < 0.05.

**Table 2:** Pre- & post-education survey descriptive statistics & comparative analysis

Outcome variables	Descriptive statistics		Paired T-tests
7 NSS-CTC self-confidence questions	Pre-Education (M, SD)	Post-Education (M, SD)	Test Statistic (*t, p*-value)
1) Monitor signs/symptoms of deteriorating cardiovascular functions	M = 3.19, SD = 0.80	M = 3.77, SD = 0.82	*t* = 3.43, *p* = 0.0021
2) Recognize symptoms/signs of secondary cardiotoxicity	M = 1.88, SD = 0.86	M = 3.65, SD = 0.80	*t* = 7.90, *p* < 0.0001
3) Recognize which chemotherapy treatments cause (reversible/irreversible heart damage)	M = 1.85, SD = 0.98	M = 3.50, SD = 0.76	*t* = 8.47, *p* < 0.0001
4) Recognize chemotherapy treatments leading to cardiovascular damage (early and late)	M = 1.92, SD = 0.74	M = 3.50, SD = 0.71	*t* = 8.47, *p* < 0.0001
5) Identify risk factors for chemotherapy-related cardiovascular complications	M = 1.78, SD = 0.91	M = 3.81, SD = 0.69	*t* = 8.69, *p* < 0.0001
6) Monitor factors that have a negative impact on cardiovascular clinical outcomes	M = 2.23, SD = 0.90	M = 3.77, SD = 0.65	*t* = 3.43, *p* < 0.0001
7) Educate patients undergoing cardiotoxic chemotherapy on how to symptoms of altered cardiovascular function	M = 2.23, SD = 0.91	M = 3.58, SD = 0.81	*t* = 6.04, *p* < 0.0001

Following the educational intervention, analysis revealed a significant increase in nursing self-efficacy across all 7 questions. Pre-education confidence levels indicated little confidence with questions 2, 3, 4, and 5 reporting mean scores below 2. Questions 6 and 7 reported a mean score of 2.23. Question 1 was the only question where some level of confidence was reported (pre-education mean score of 3.19). Post-educational intervention, the mean scores for all 7 questions ranged from 3.50 to 3.81, denoting a substantial improvement in how nurses reported their self-efficacy in recognizing cancer treatment-related cardiotoxicity.

The pre- and post-education surveys included one self-efficacy yes or no question (Table 3). This question asked if RNs thought they could recognize a patient with symptoms of cancer therapy-related cardiotoxicity. Before participating in the educational intervention 2 RNs (7.7%) answered “yes” in their ability to recognize such symptoms. Whereas 24 RNs (92.3%) did not believe they had the capacity to recognize such symptoms. Following the educational intervention all 26 RNs (100%) answered “yes” in their ability to detect symptoms of cancer therapy-related cardiotoxicity. Zero RNs (0%) answered “no”.

**Table 3:** Self-efficacy question participant responses before and after Educational intervention

Pre- and Post-Intervention Self-Efficacy Question	Yes	No
	N (%)	N (%)
Before taking part in this education, do you think you can recognize a patient with symptoms of cancer therapy-related cardiotoxicity?	2 (7.7%)	24 (92.3%)
After taking part in this education, do you think you can recognize a patient with symptoms of cancer therapy-related cardiotoxicity?	26 (100%)	0 (0%)

## Discussion

A bachelor’s degree in nursing emerged as the most common education level among participants, indicating a high-level of formal education. Zero nurses reported having prior oncology nursing experience, this is a critical data point given the context of the study. The majority of the respondents were employed in the CCU indicating a focused expertise in cardiac care within the participant group.

Comparative analysis of pre-and post-education surveys underscored the effectiveness of the targeted educational intervention. The results showed the self-efficacy of cardiac nurses with no previous oncology experience can progress from little confidence towards confident after one educational intervention.

The results clearly highlight the effectiveness of the targeted educational program, with a marked improvement in the self-efficacy scores from pre- to post-intervention across all measures. This improvement in self-confidence not only supports nurses’ ability to detect and manage cardiotoxic events earlier but also underscores the need for specialized training within cardiac nursing practice. This is particularly essential in settings where patients have multiple high risk cardiac factors placing them at a heightened risk of developing cancer therapy-related cardiotoxicity.

### Contribution to cardio-oncology nursing practice

Given that currently no formal academic cardio-oncology nurse training program or certification exists, there is clearly more work to be done on the role of nurses in cardio-oncology care. The results underscore the need for comprehensive training programs in cardio-oncology nursing. While previous studies have noted the general efficacy of educational interventions for enhancing clinical skills, this study is distinct as it specifically measures changes in self-efficacy related to cardiotoxicity—a key area that has not been sufficiently addressed in nursing education. By focusing on cardiac nurses without prior oncology experience, the intervention bridges a critical gap in knowledge, equipping them with the necessary skills to improve patient outcomes significantly.

### Link between self-efficacy and clinical competence

The increase in self-efficacy observed post-intervention suggests that educational programs can effectively enhance the competence of nurses, even in highly specialized areas like cardio-oncology. This link supports the theoretical framework proposed by Bandura, where self-efficacy serves as a foundation for higher competence in performing complex clinical tasks. Higher self-efficacy in recognizing cardiotoxicity symptoms can lead to more proactive and timely interventions, potentially reducing the severity of cardiotoxic outcomes in patients undergoing cancer treatment.

### Implications for healthcare systems

This research underscores the necessity for healthcare systems to embed cardiotoxicity recognition training for nurses. As cardiotoxicity in cancer patients continues to rise, integrating cardio-oncology into healthcare services becomes imperative [9]. Empowering nurses with education to detect early signs of cardiotoxicity is a strategic move to curtail the long-term morbidity and mortality linked to cancer treatments [8]. Such specialized training fosters interdisciplinary collaboration, enhancing the synergy between nurses and cardio-oncology experts. It also highlights the critical role of continuous nursing education within healthcare systems in elevating patient outcomes and overall healthcare quality [1].

### Implications for healthcare policy

The findings of this study highlight the lack of and advocates for cardiotoxicity recognition training for nurses. Enacting policies requiring consistent training and assessment of nursing competencies in specialized areas ensure nurses are adeptly equipped to address complex patient needs [6]. Policies should also cultivate an environment conducive to continuous professional development, providing the necessary support for nurse’s educational advancement [12]. It is imperative for health care policies to acknowledge and address the unique challenges faced by cardiac nurses by enhancing self-efficacy in their roles.

### Implications for healthcare economics

Investing in nursing education for cardiotoxicity recognition can lead to significant financial benefits for both hospitals and patients, including cost savings. Potential cost savings are derived from the reduced necessity for cardiotoxicity-related treatments and shorter hospital stays [14]. Focused educational interventions, such as the one in this study, can potentially reduce long-term costs associated with managing cardiotoxicity [8]. Wong et al. [13] performed an assessment of health care costs associated with adverse cardiac events in 412,005 patients with cancer. Adverse cardiac events during treatment episodes for cancer were frequent [13]. Common cardiotoxicity complications have high costs per treatment episode. The cost per episode for common cardiotoxic complications include treatment for hypertension at $28,983, chest pain/angina at $20,081, thromboembolic events at $26,080, arrhythmias at $25,232, heart failure at $26,348, and hypotension at $21,378 [13]. Implementing educational interventions focused on nurse competence in early recognition of cardiotoxicity potentially could save both patients and hospital systems money by avoiding adverse cardiac events.

### Implications for nursing practice

A cardiac nurse is a crucial member of the interdisciplinary cardio-oncology team. A patient receiving potentially cardiotoxic cancer treatment may first present signs of cardiotoxicity in the presence of an RN on a cardiac unit, rather than an oncology unit. This is the first known study that generated data concerning the impact of an educational intervention on the early cardiotoxicity recognition by the cardiac nurse population. Enhanced early recognition of cardiotoxic symptoms by nurses is crucial in preventing further deterioration and ensuring timely treatment [12]. Intervening through cancer therapy stopping, pausing, adjusting cardiotoxic cancer therapy and adding cardio-protective medications is critical to prevention of cardiotoxicity progression. Increased self-efficacy among nurses leads to necessary consultations with cardio-oncology providers [8]. Educational programs tailored to the unique requirements of nurses in cardiac settings are vital. Additionally, improving nurse competence in the early recognition of cardiotoxicity can aid hospitals during a Joint Commission accreditation visit, help to achieve Magnet Status, and inform the development of cardio-oncology programs.

### Limitations

The study presents valuable insights, but has limitations. The use of self-reported measures for self-efficacy could introduce subjective biases. Excluding certain questions from the NSS-CTC could limit the survey comprehensiveness. Modifications were made to the NSS-CTC by selectively removing certain items to tailor the scale specifically to cardiac nursing contexts, which may impact its original validation metrics. Further research is needed to reassess the psychometric properties of this adapted version to ensure its continued reliability and validity in this specialized setting. Environmental factors in the clinical setting, such as interruptions, could impact the effectiveness of the educational intervention. Additionally, each participant attending a single 30-minute session encompassing both the pre-education survey and post-education survey limits the study’s ability to assess long-term knowledge retention or sustained changes in practice. This format restricts the evaluation of enduring educational impacts beyond the immediate post-intervention period.

## Conclusion

The findings of this study highlight the need for cardio-oncology nursing education to provide safe care to cancer patients with cardiovascular issues. The lack of education for cardiac nurses against the backdrop of increasing cardiotoxicity in cancer patients showcases the essential need for cardiac nurse early symptom recognition education. This study demonstrates the effectiveness of an educational intervention on improving cardiac nurses self-efficacy. The impact will extend beyond nursing practice. Healthcare policies, economics, and systems can be influenced by the outcomes of this study. This study paves the way for the importance of setting aside resources for education surrounding cardiotoxicity.

### Knowledge translation

1. A cardiac nurse is a crucial member of the interdisciplinary cardio-oncology team.
2. A patient may first present signs of cardiotoxicity in the presence of an RN on a cardiac unit, rather than an oncology unit.
3. The self-efficacy of cardiac nurses with no previous oncology experience can progress from little confidence towards confident after one education intervention.

## Electronic supplementary material

Below is the link to the electronic supplementary material.

Supplementary Material 1

Supplementary Material 2

## Data Availability

No datasets were generated or analysed during the current study.
