# Supplementary material for: Enhancing nurse competence in early recognition of cardiotoxicity
Source: Cardiooncology. 2024 Sep 14;10:62. doi: 10.1186/s40959-024-00261-x (PMC11401397; doi:10.1186/s40959-024-00261-x)
Supplement: Supplementary file 1 — Supplementary Material 1 [file 40959_2024_261_MOESM1_ESM.pptx]

## Slide 1
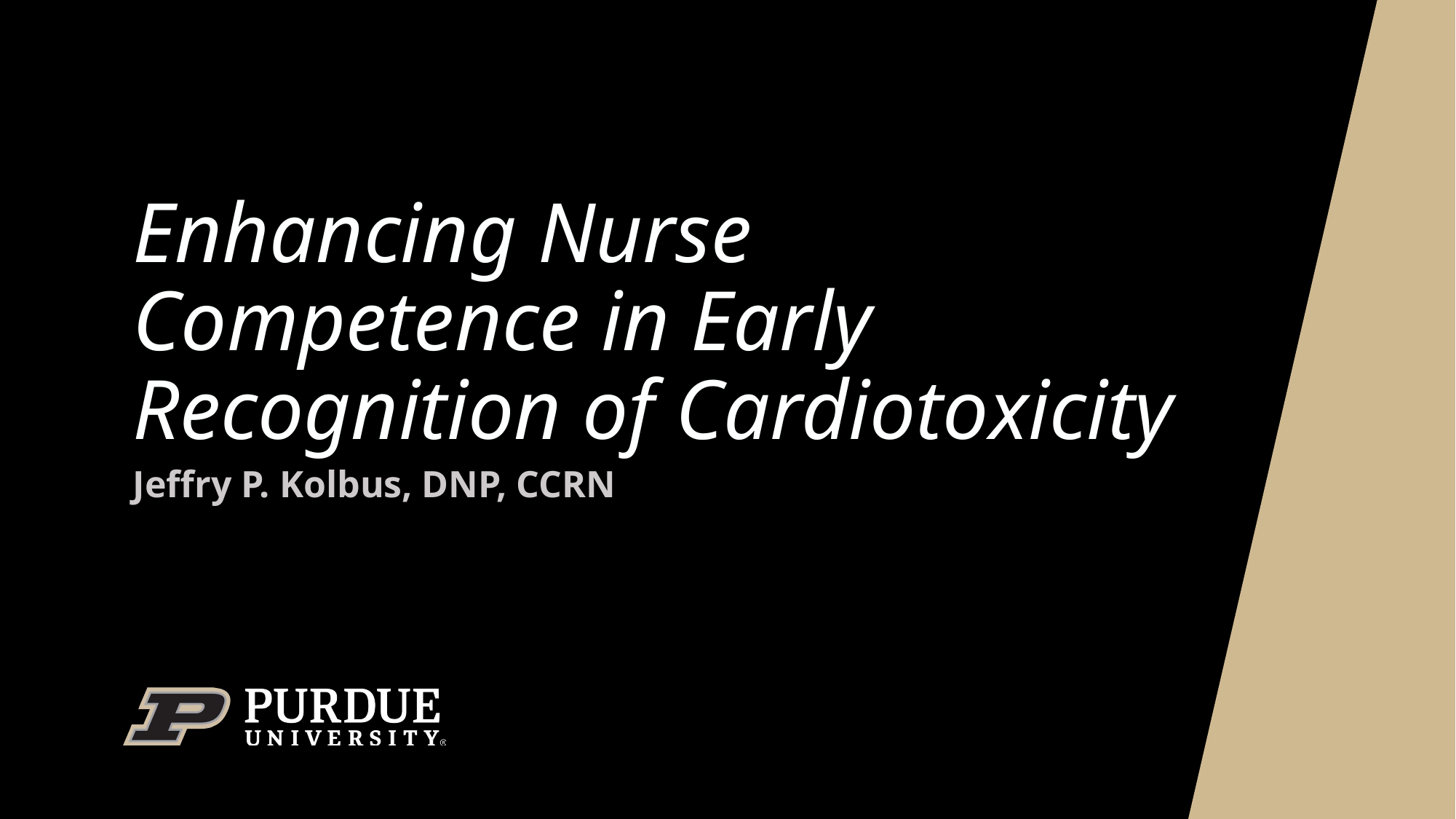

# Enhancing Nurse Competence in Early Recognition of Cardiotoxicity
Jeffry P. Kolbus, DNP, CCRN

## Slide 2
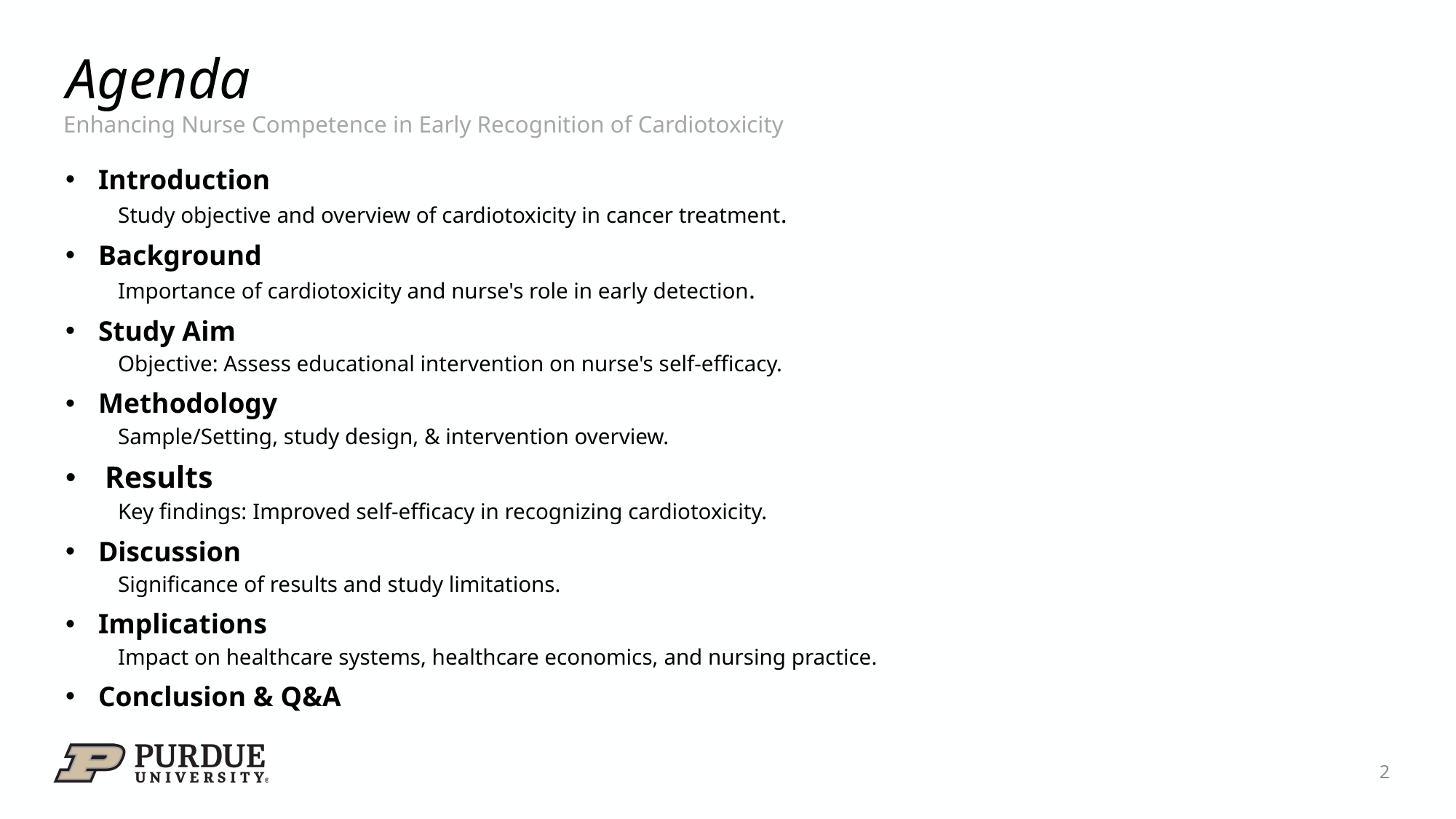

# Agenda
Enhancing Nurse Competence in Early Recognition of Cardiotoxicity
Introduction
Study objective and overview of cardiotoxicity in cancer treatment.
Background
Importance of cardiotoxicity and nurse's role in early detection.
Study Aim
Objective: Assess educational intervention on nurse's self-efficacy.
Methodology
Sample/Setting, study design, & intervention overview.
Results
Key findings: Improved self-efficacy in recognizing cardiotoxicity.
Discussion
Significance of results and study limitations.
Implications
Impact on healthcare systems, healthcare economics, and nursing practice.
Conclusion & Q&A
2

## Slide 3
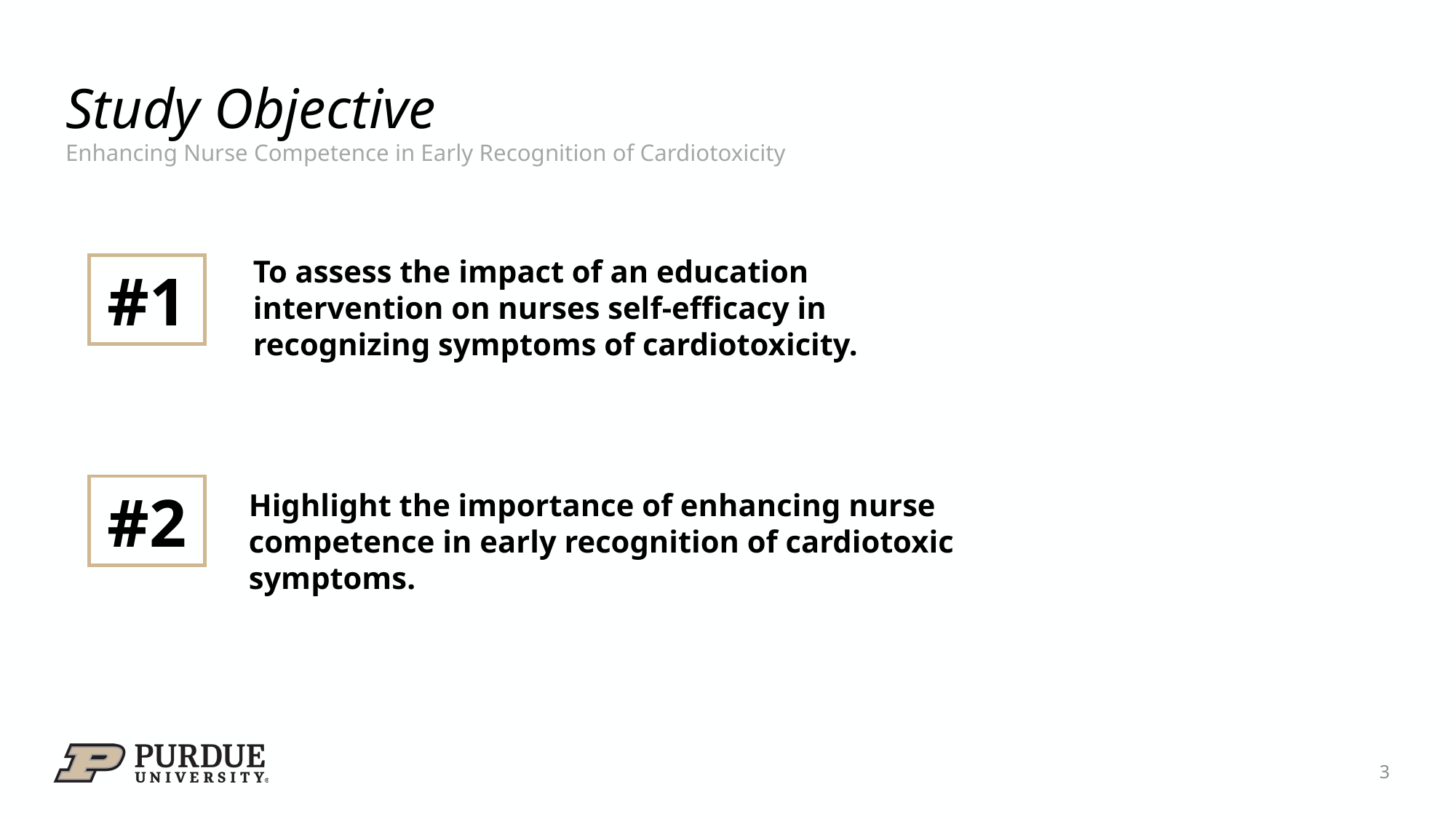

# Study Objective
Enhancing Nurse Competence in Early Recognition of Cardiotoxicity
To assess the impact of an education intervention on nurses self-efficacy in recognizing symptoms of cardiotoxicity.
#1
#2
Highlight the importance of enhancing nurse competence in early recognition of cardiotoxic symptoms.
3

## Slide 4
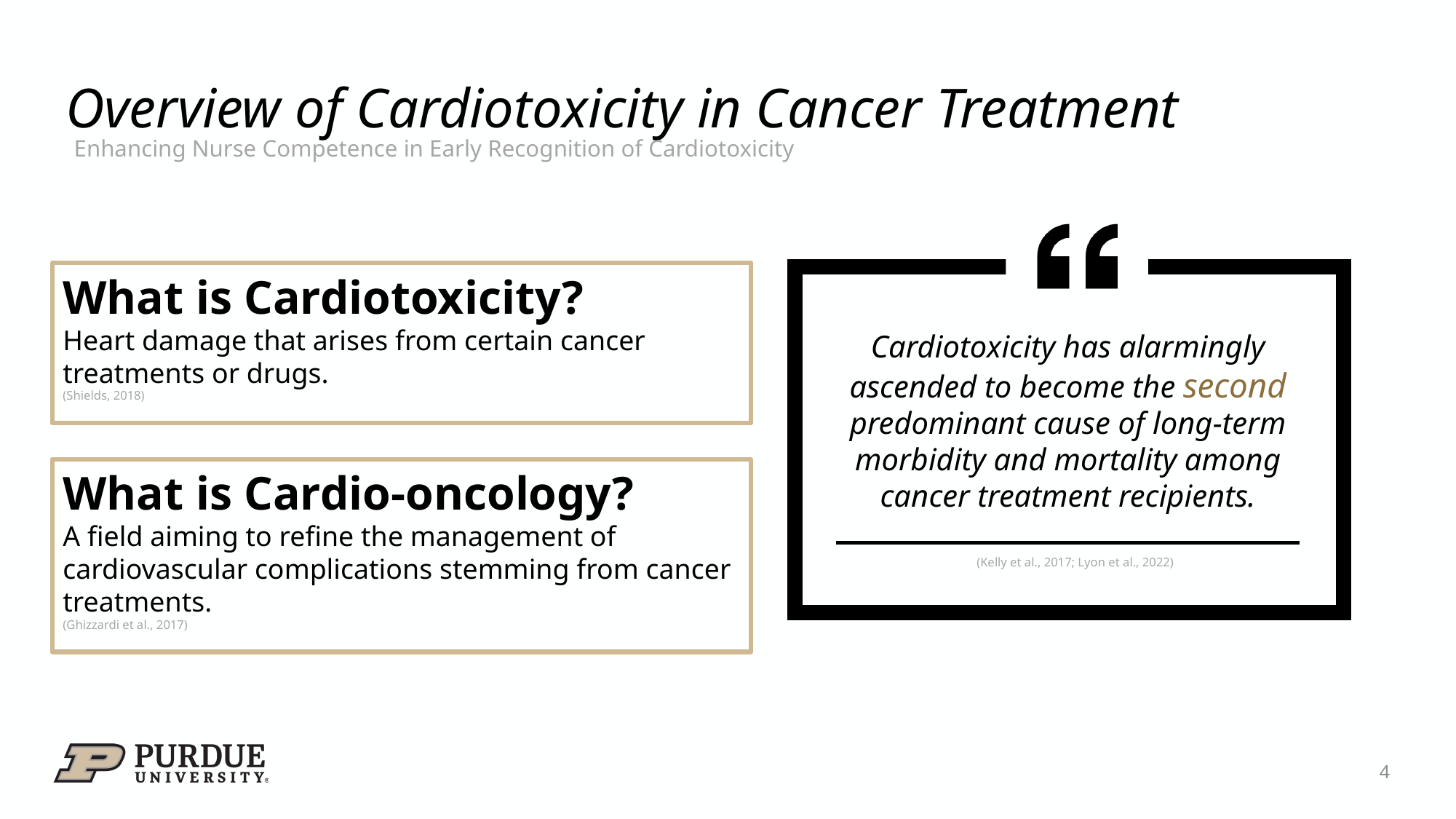

# Overview of Cardiotoxicity in Cancer Treatment
Enhancing Nurse Competence in Early Recognition of Cardiotoxicity
Cardiotoxicity has alarmingly ascended to become the second predominant cause of long-term morbidity and mortality among cancer treatment recipients.
(Kelly et al., 2017; Lyon et al., 2022)
What is Cardiotoxicity?
Heart damage that arises from certain cancer treatments or drugs.
(Shields, 2018)
What is Cardio-oncology?
A field aiming to refine the management of cardiovascular complications stemming from cancer treatments.
(Ghizzardi et al., 2017)
4

## Slide 5
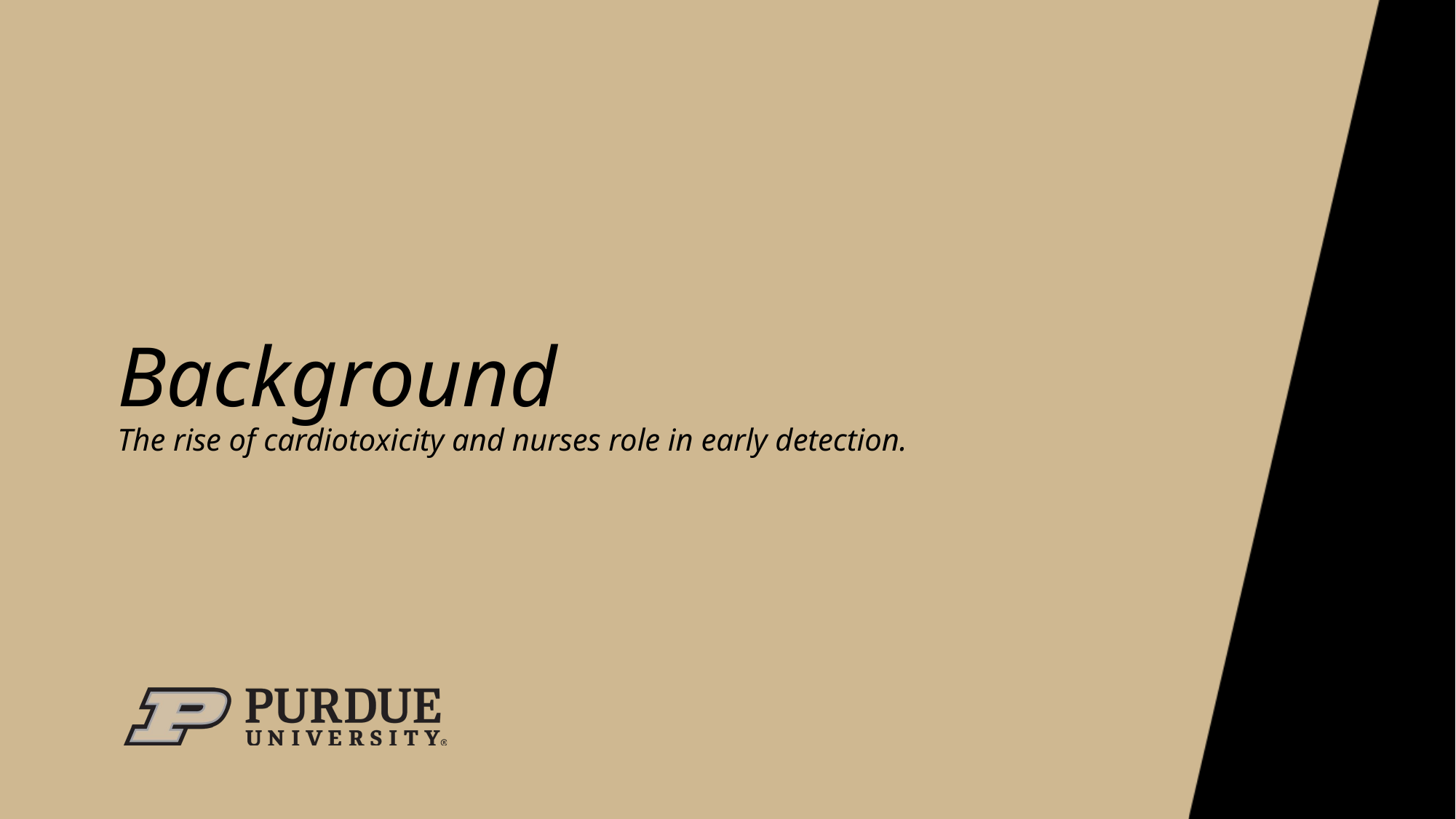

# Background The rise of cardiotoxicity and nurses role in early detection.

## Slide 6
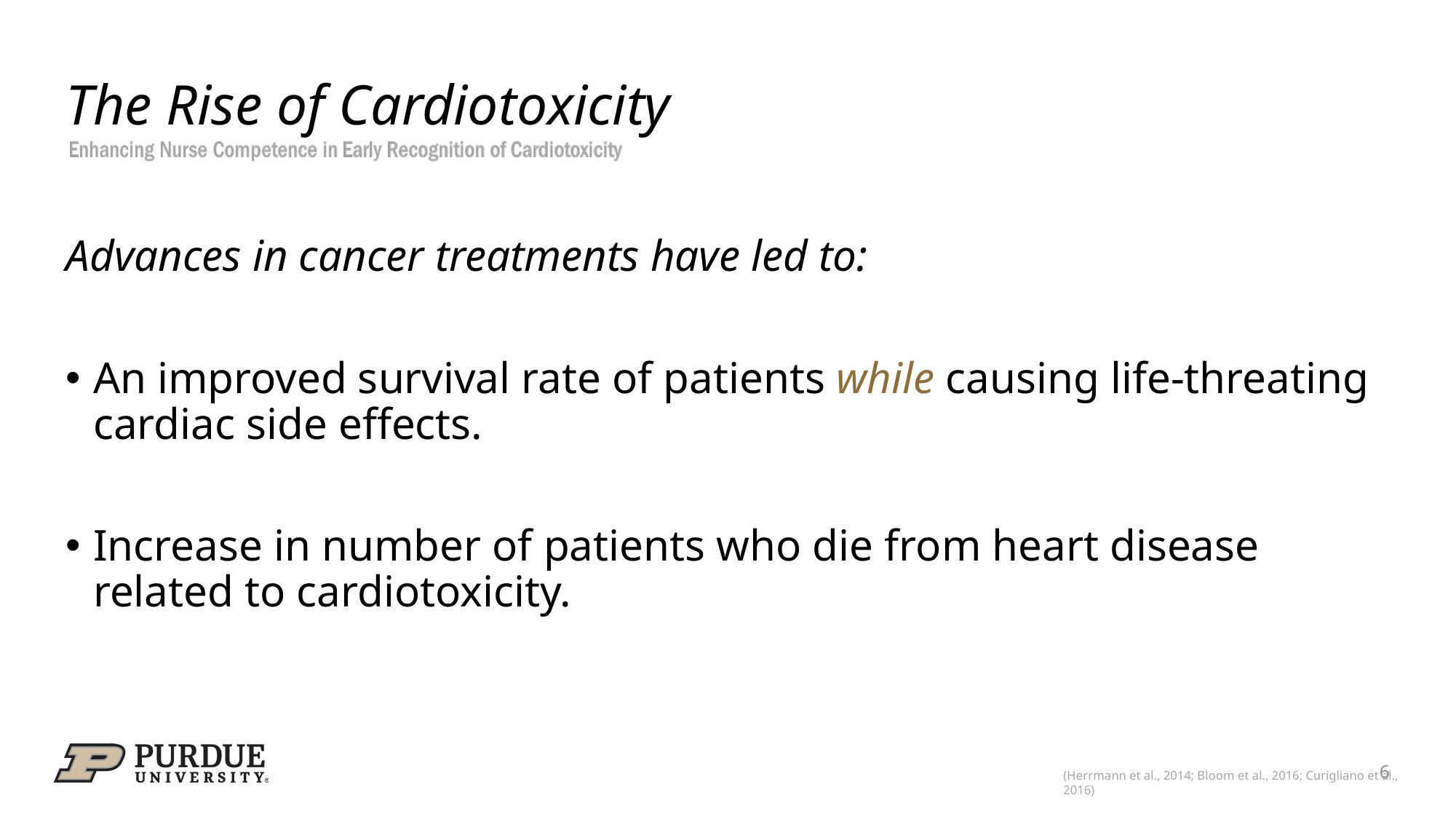

# The Rise of Cardiotoxicity
Advances in cancer treatments have led to:
An improved survival rate of patients while causing life-threating cardiac side effects.
Increase in number of patients who die from heart disease related to cardiotoxicity.
6
(Herrmann et al., 2014; Bloom et al., 2016; Curigliano et al., 2016)

## Slide 7
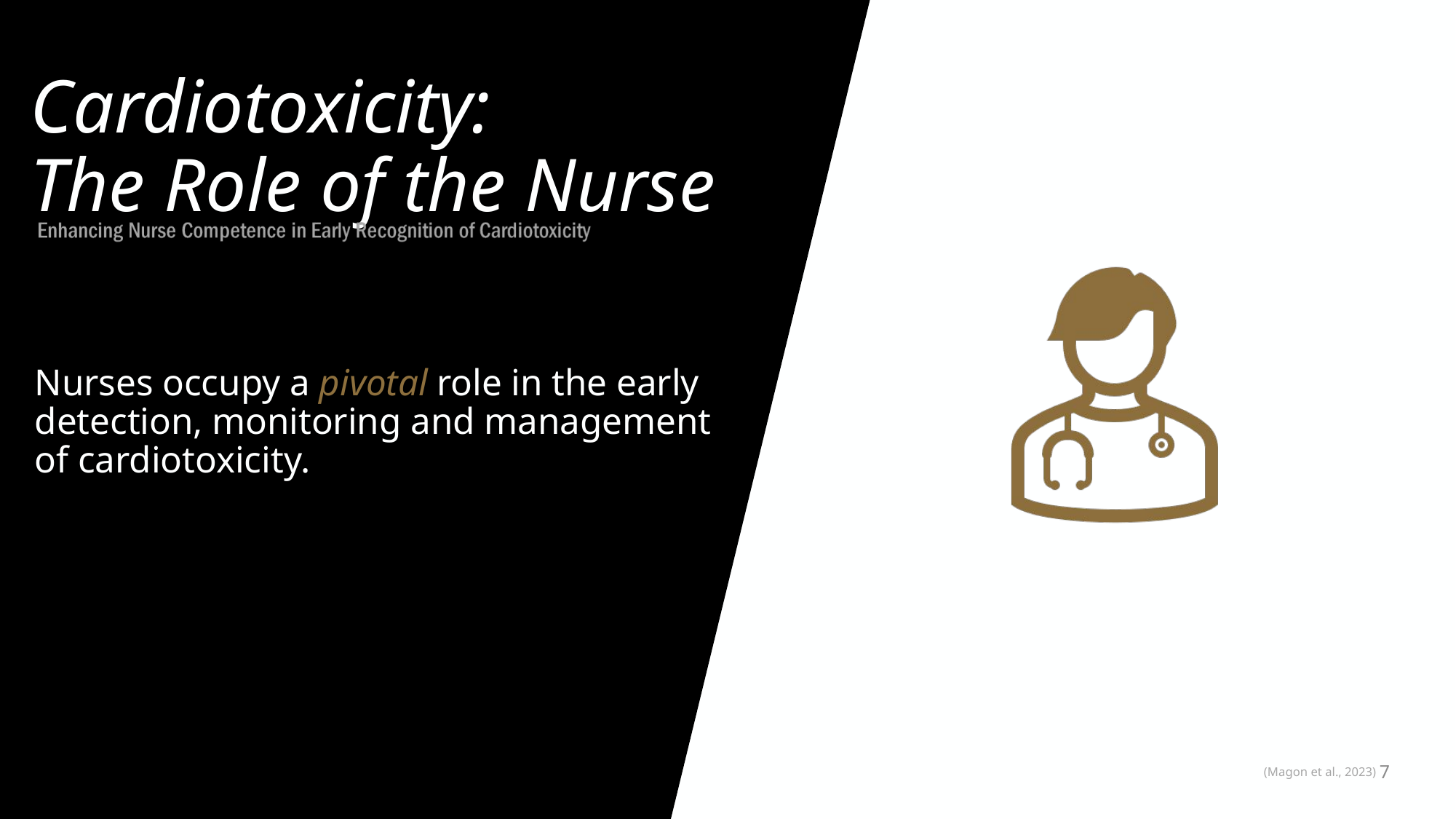

# Cardiotoxicity: The Role of the Nurse
Nurses occupy a pivotal role in the early detection, monitoring and management of cardiotoxicity.
7
(Magon et al., 2023)

## Slide 8
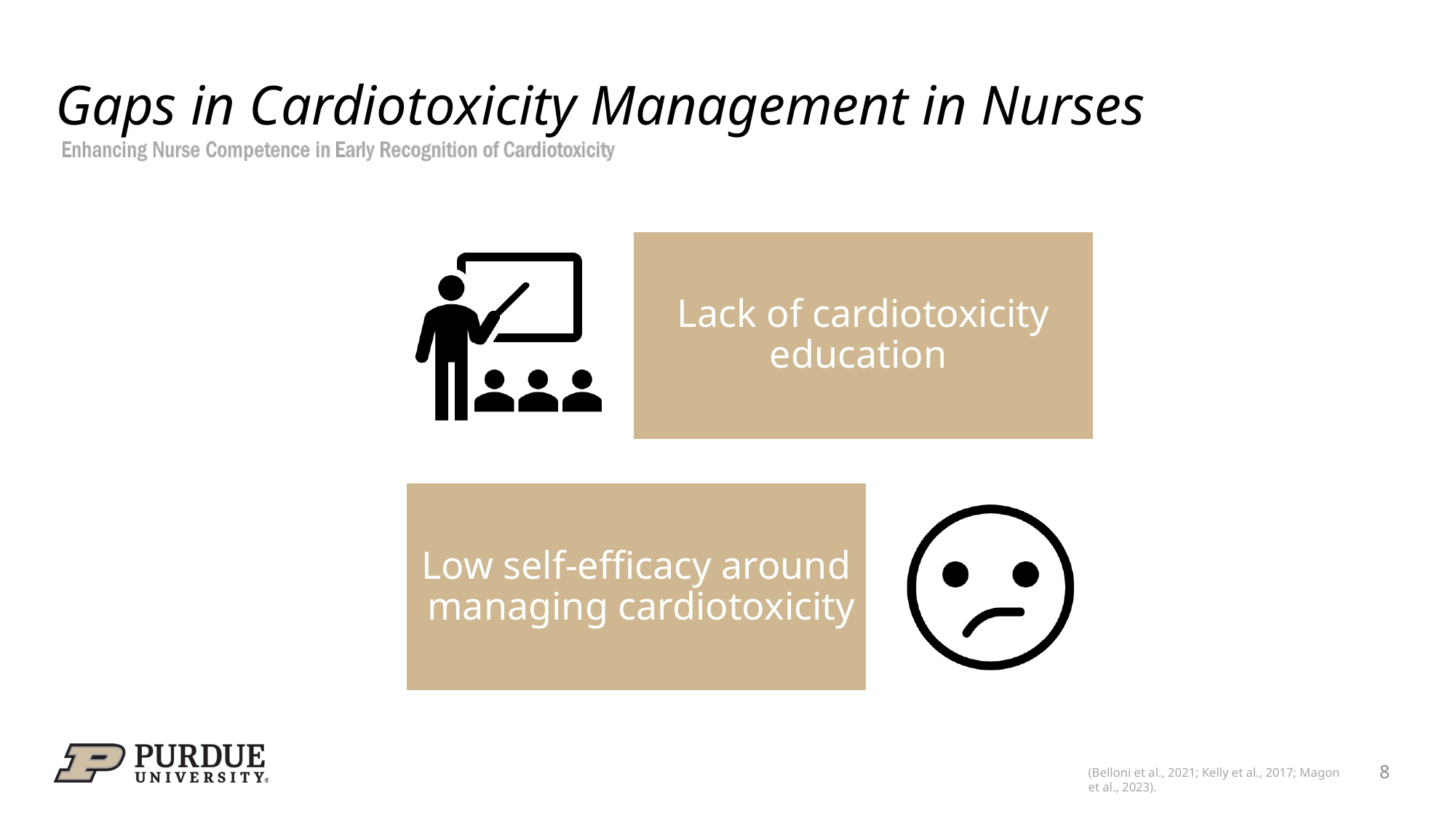

# Gaps in Cardiotoxicity Management in Nurses
Lack of cardiotoxicity education
Low self-efficacy around managing cardiotoxicity
8
(Belloni et al., 2021; Kelly et al., 2017; Magon et al., 2023).

## Slide 9
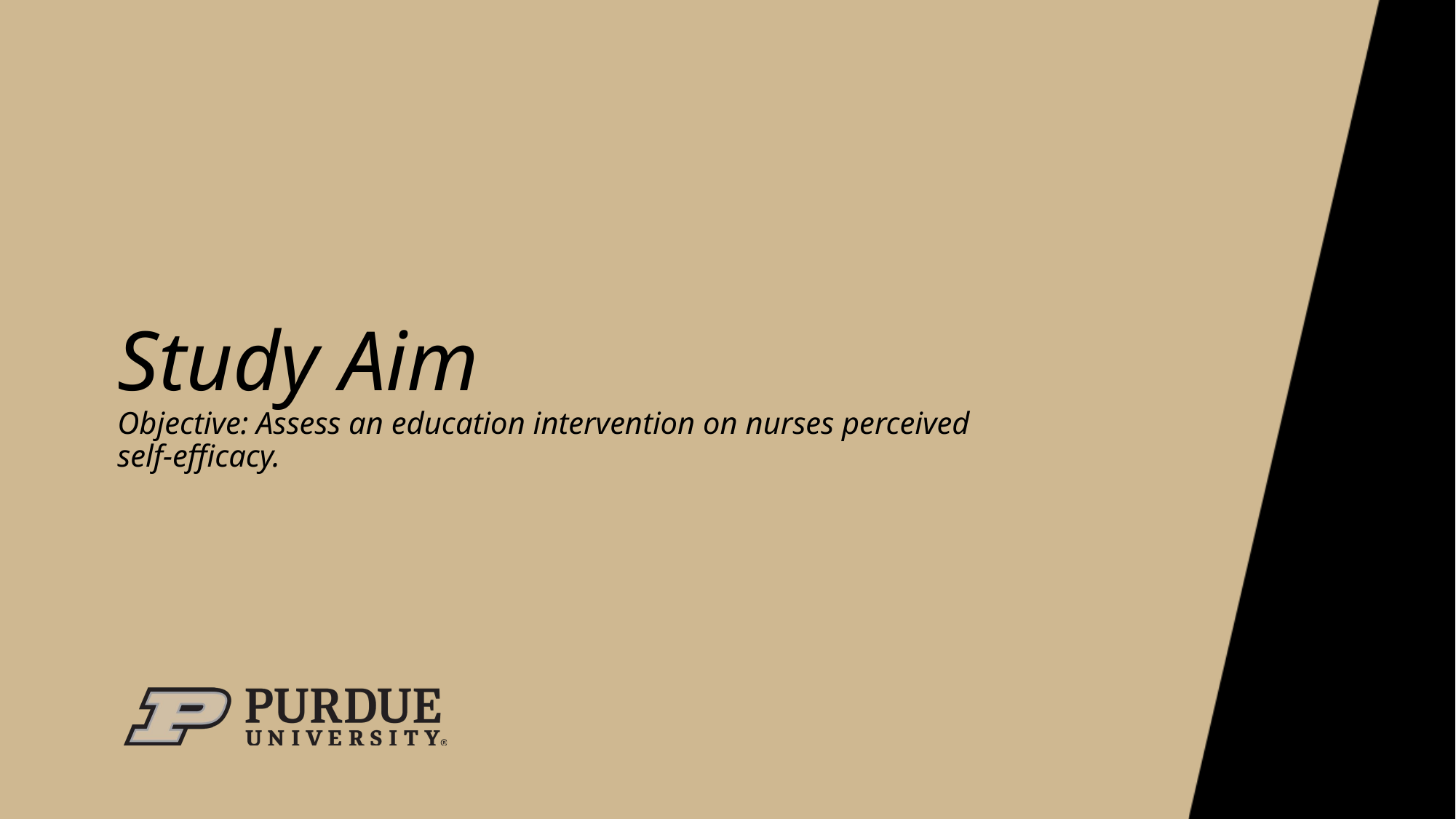

# Study Aim Objective: Assess an education intervention on nurses perceived self-efficacy.

## Slide 10
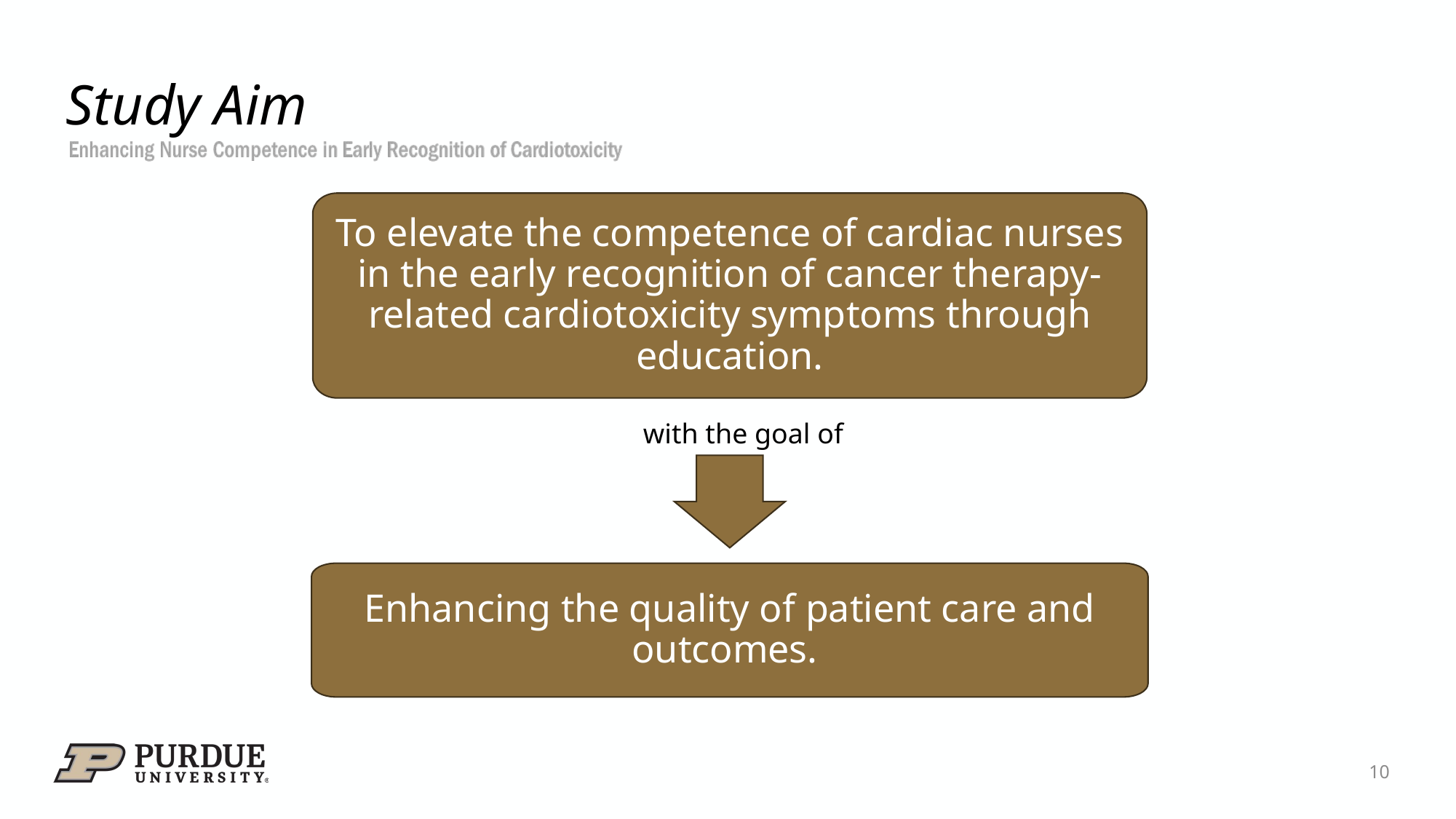

# Study Aim
To elevate the competence of cardiac nurses in the early recognition of cancer therapy-related cardiotoxicity symptoms through education.
with the goal of
Enhancing the quality of patient care and outcomes.
10

## Slide 11
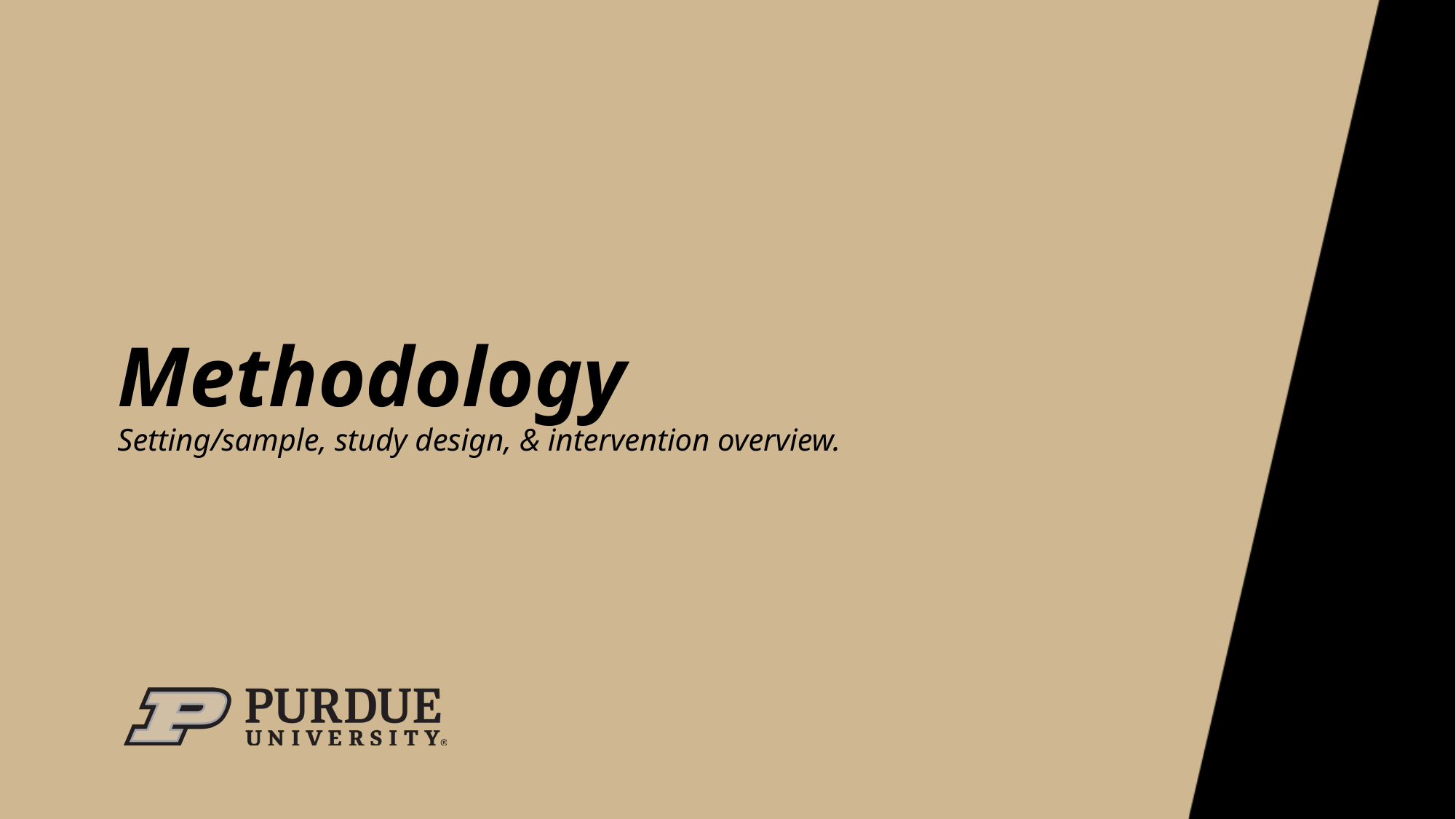

# Methodology Setting/sample, study design, & intervention overview.

## Slide 12
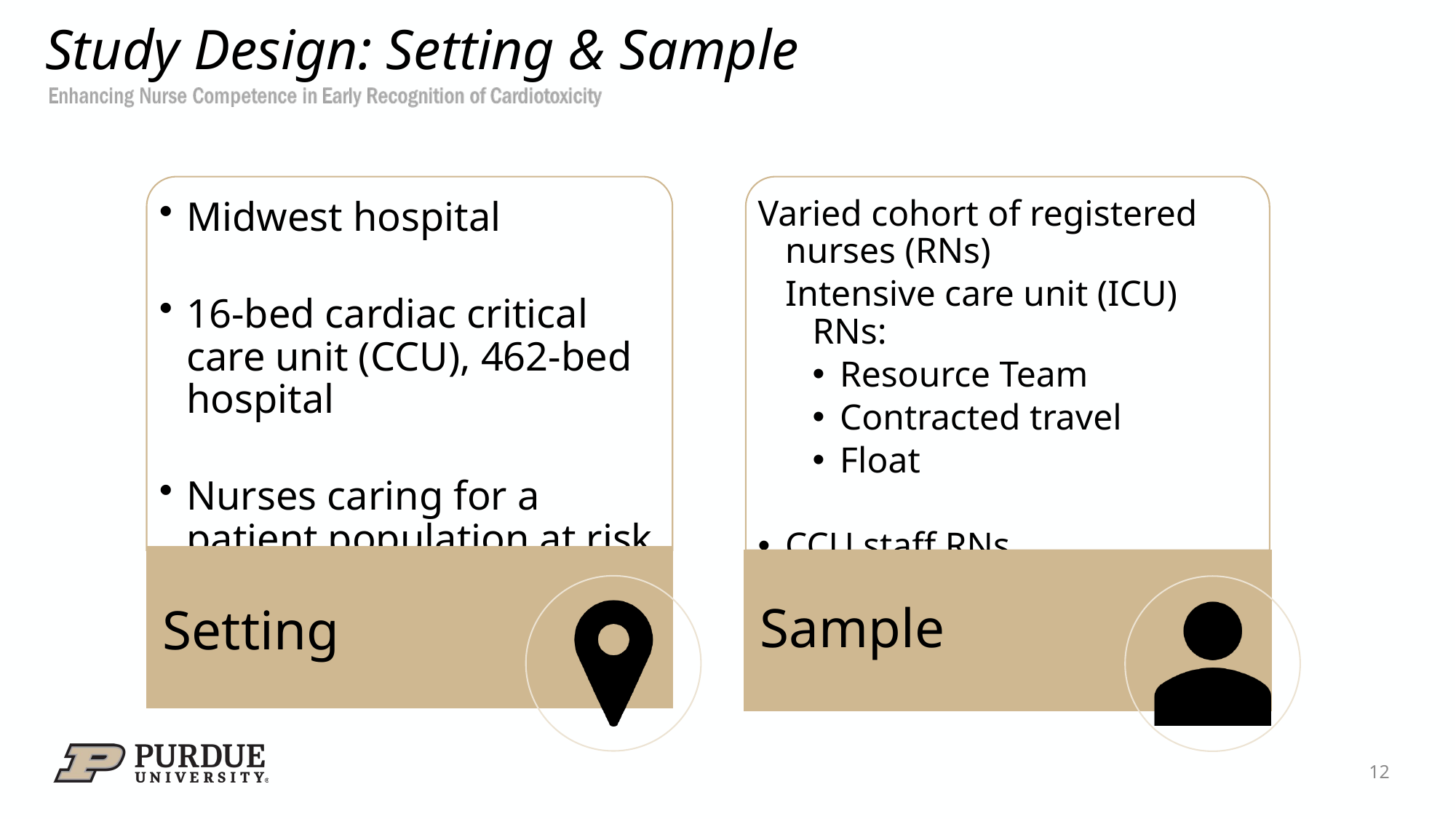

# Study Design: Setting & Sample
12

## Slide 13
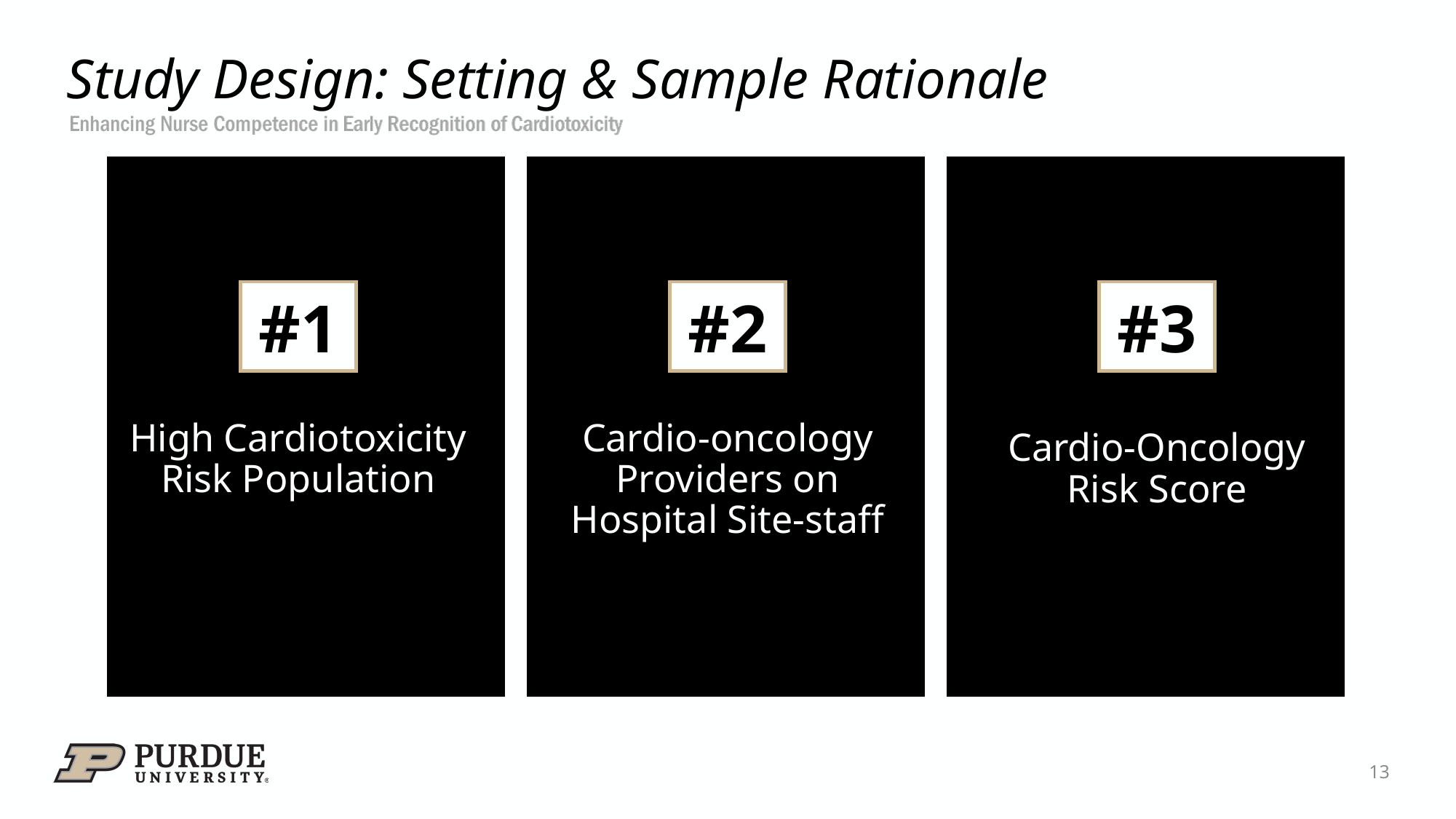

# Study Design: Setting & Sample Rationale
#1
#2
#3
High Cardiotoxicity Risk Population
Cardio-oncology Providers on Hospital Site-staff
Cardio-Oncology Risk Score
13

## Slide 14
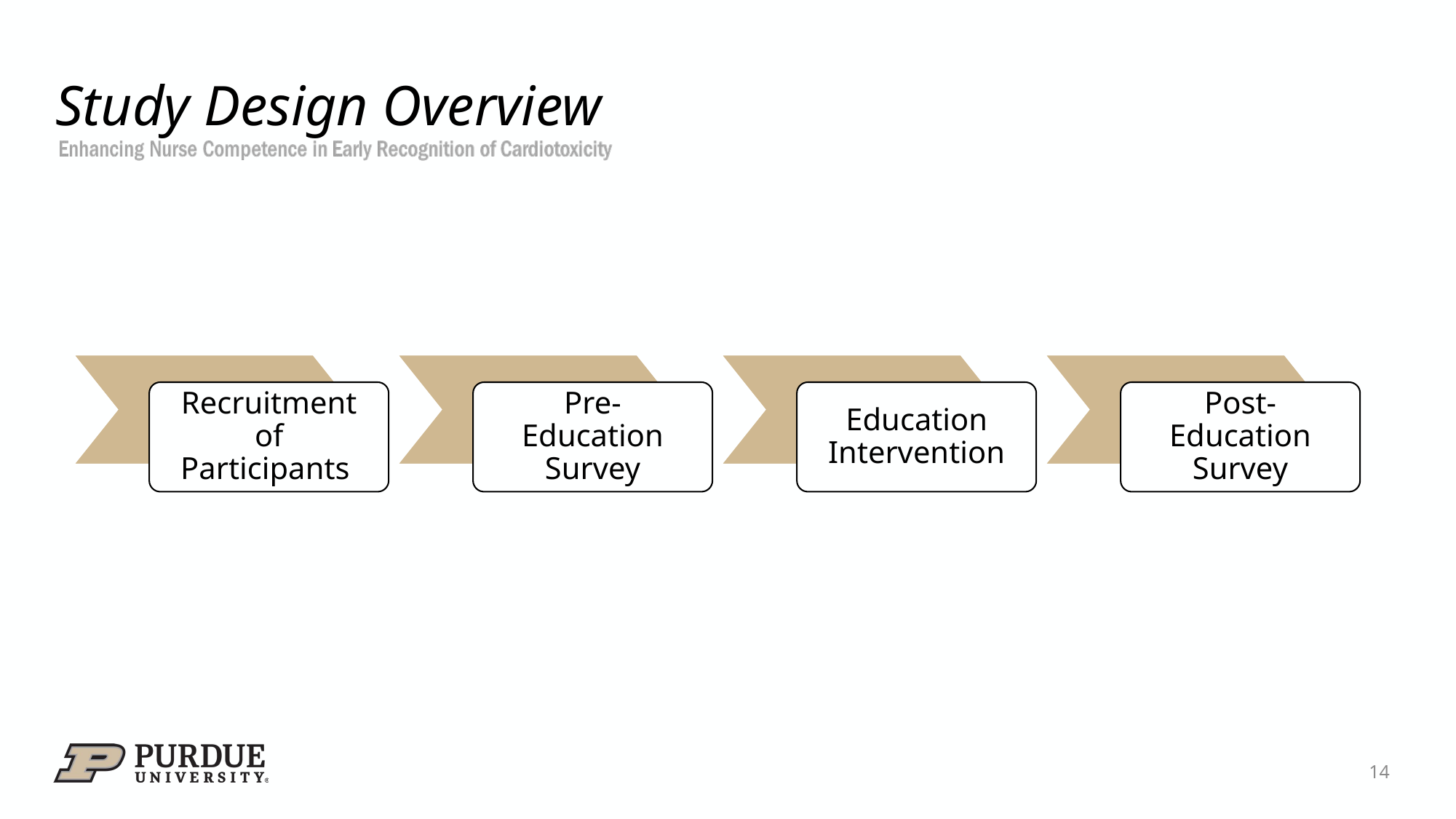

# Study Design Overview
Recruitment of Participants
Pre-Education Survey
Education Intervention
Post-Education Survey
14

## Slide 15
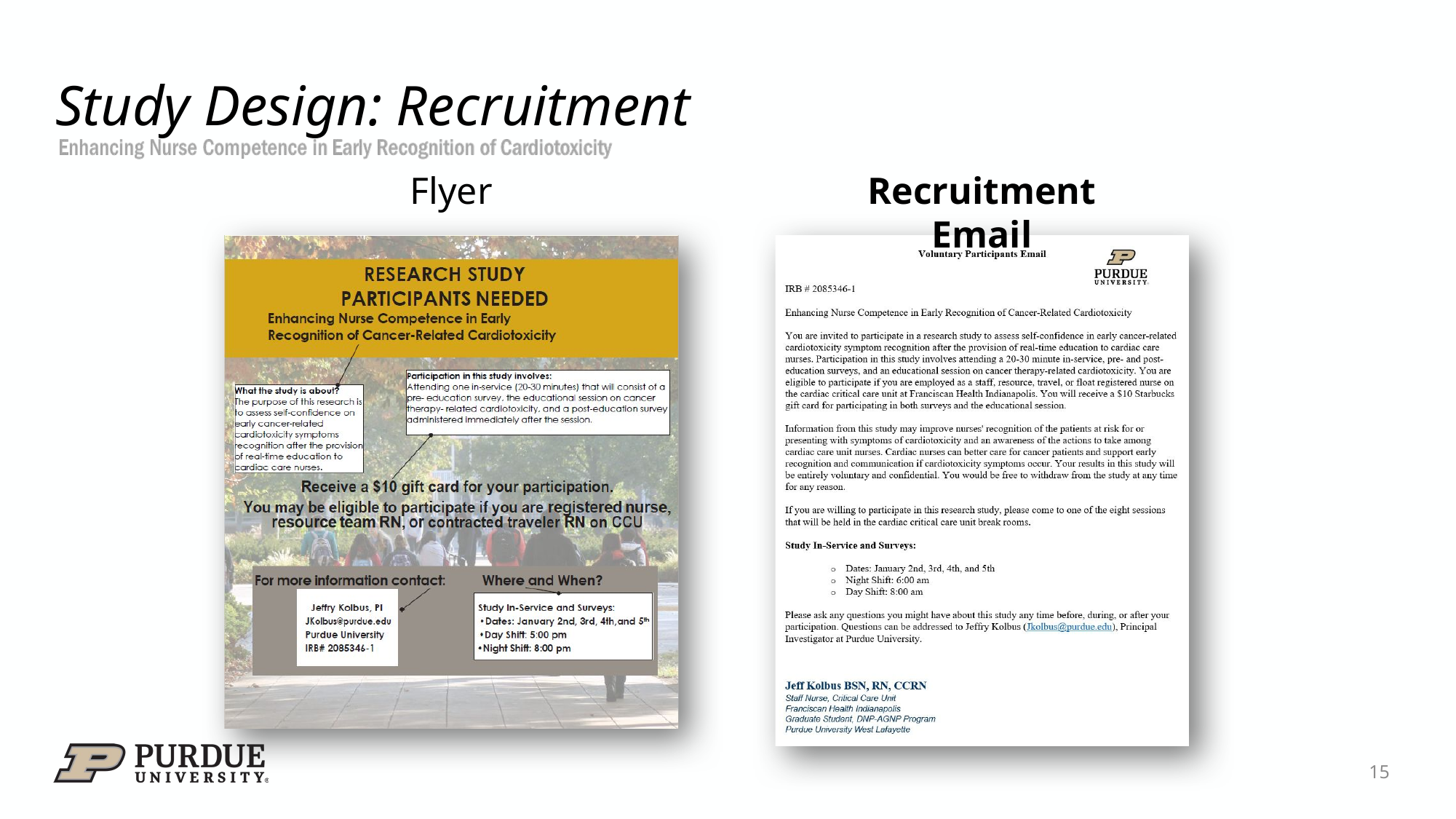

# Study Design: Recruitment
Flyer
Recruitment Email
15

## Slide 16
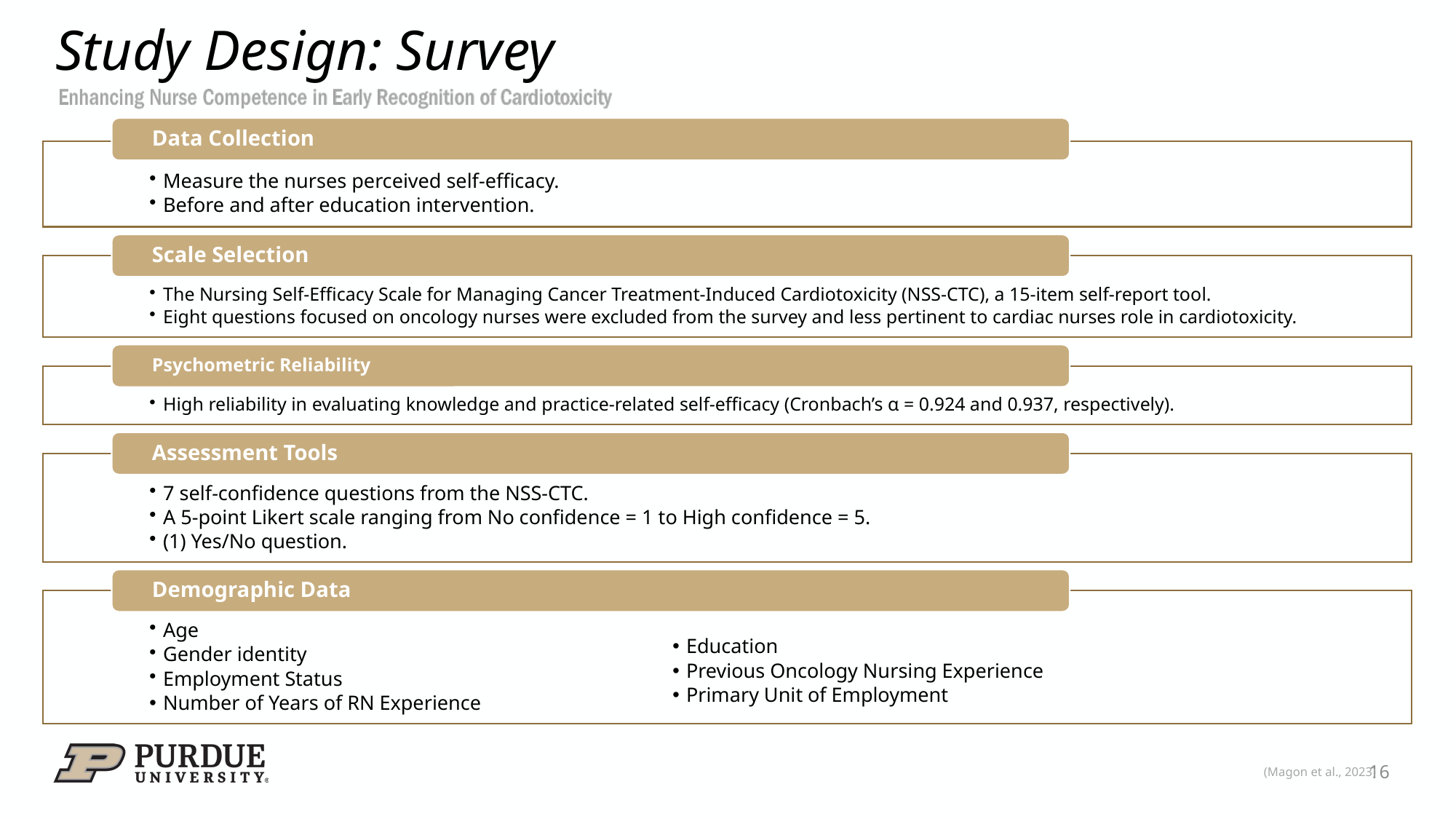

# Study Design: Survey
Education
Previous Oncology Nursing Experience
Primary Unit of Employment
16
(Magon et al., 2023)

## Slide 17
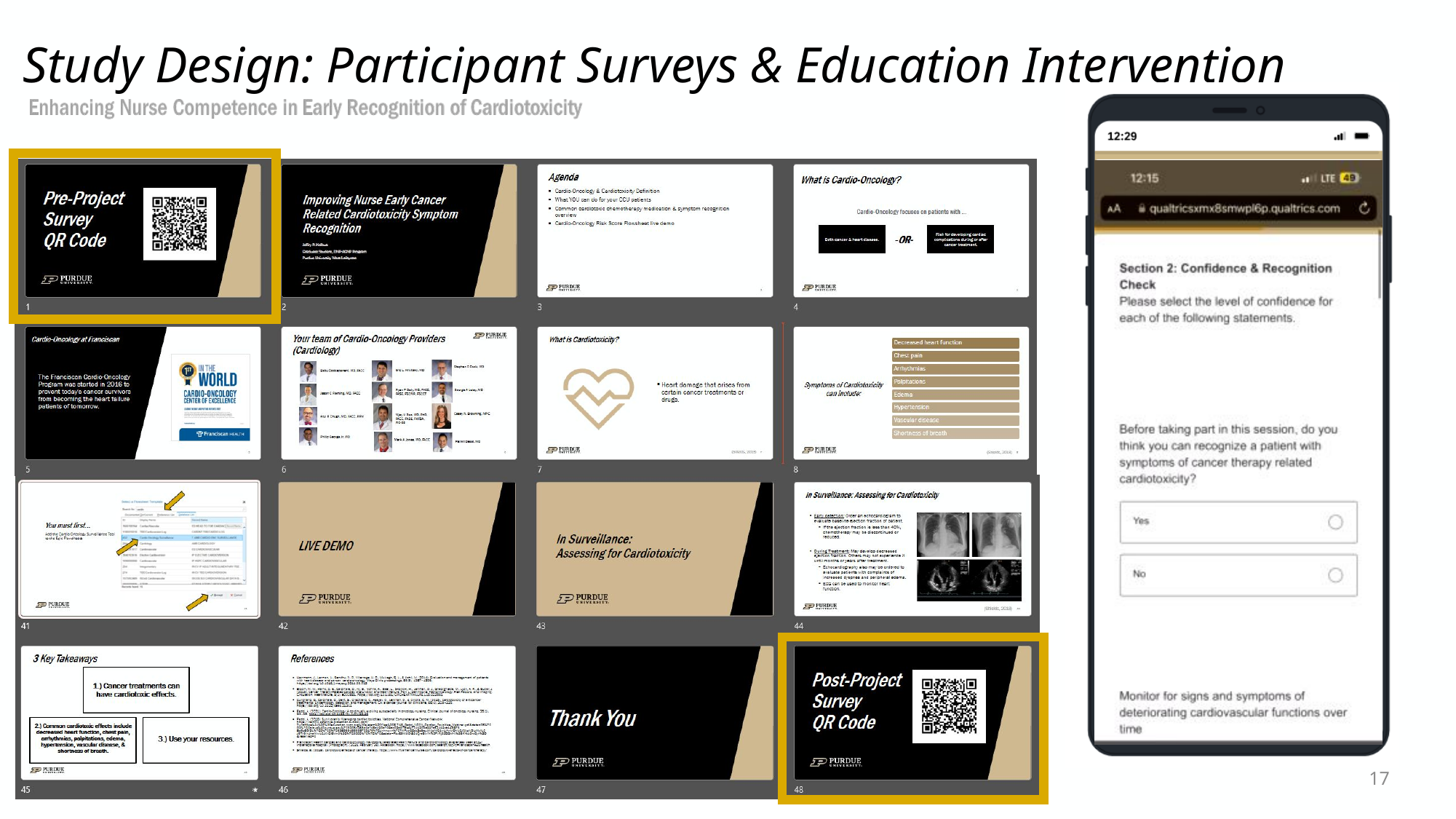

# Study Design: Participant Surveys & Education Intervention
17

## Slide 18
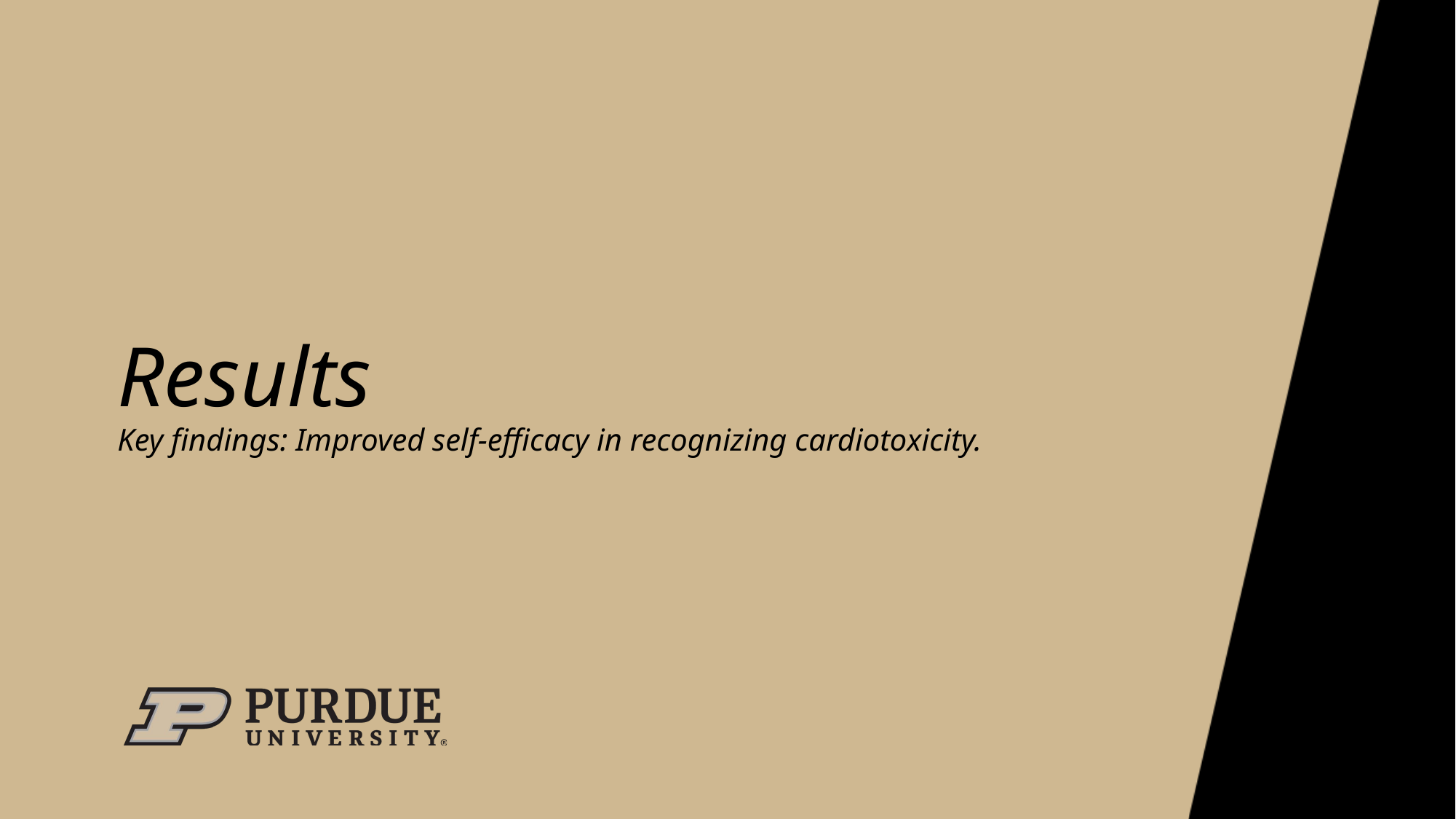

# Results Key findings: Improved self-efficacy in recognizing cardiotoxicity.

## Slide 19
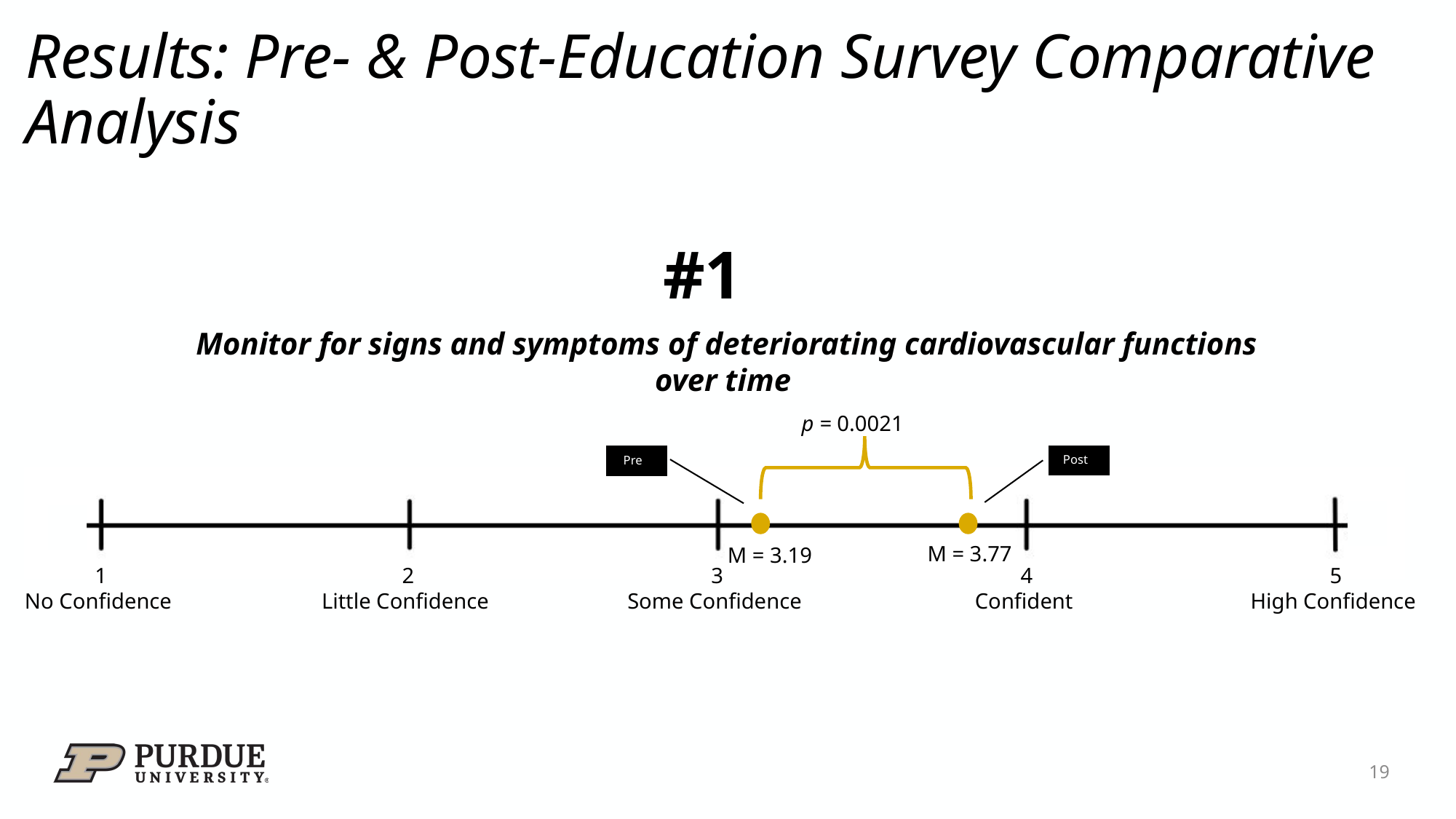

# Results: Pre- & Post-Education Survey Comparative Analysis
#1
Monitor for signs and symptoms of deteriorating cardiovascular functions over time
p = 0.0021
Pre
Post
1
No Confidence
2
Little Confidence
3
Some Confidence
4
Confident
5
High Confidence
M = 3.77
M = 3.19
19

## Slide 20
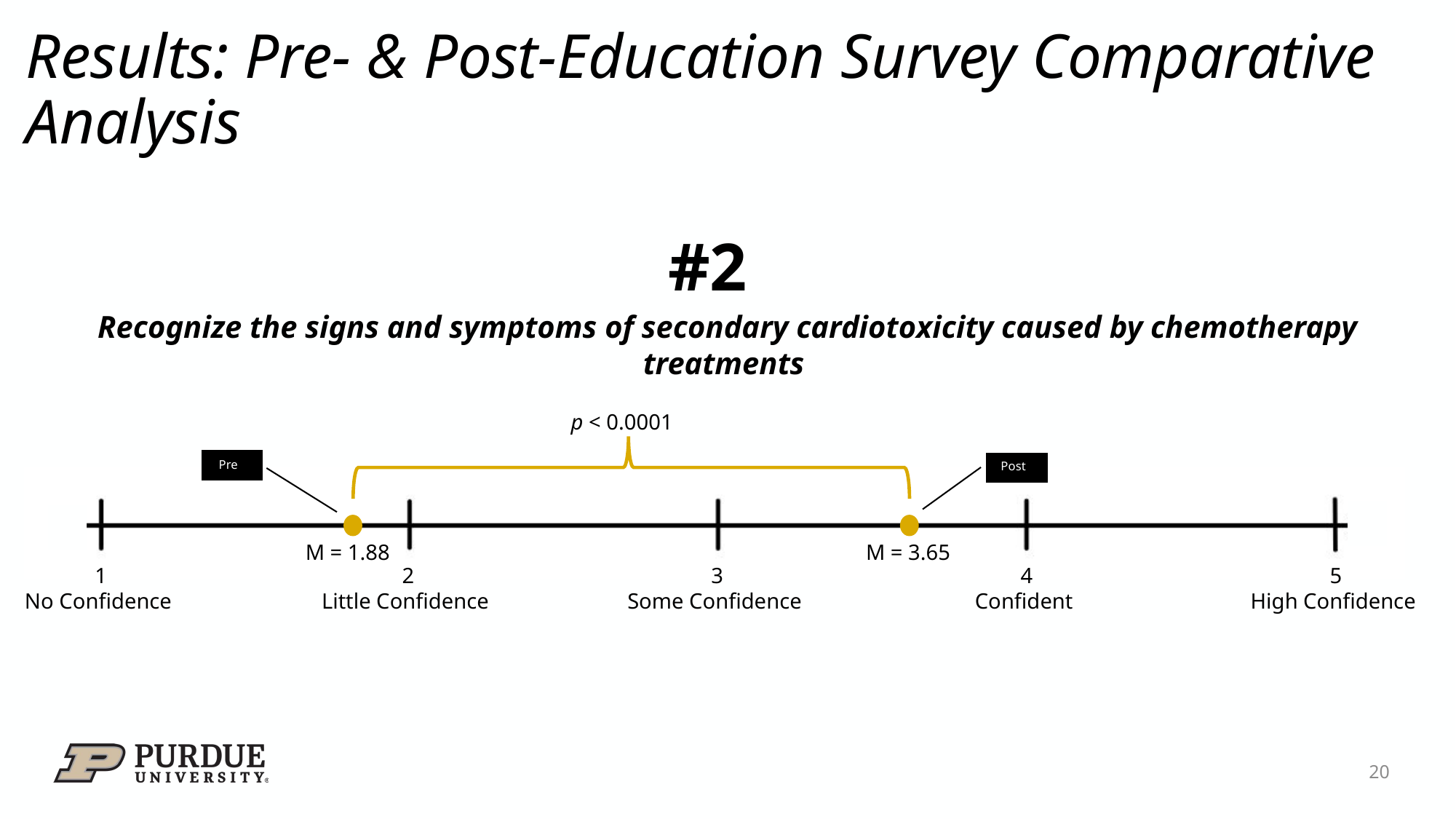

# Results: Pre- & Post-Education Survey Comparative Analysis
#2
Recognize the signs and symptoms of secondary cardiotoxicity caused by chemotherapy treatments
p < 0.0001
Pre
Post
1
No Confidence
2
Little Confidence
3
Some Confidence
4
Confident
5
High Confidence
M = 1.88
M = 3.65
20

## Slide 21
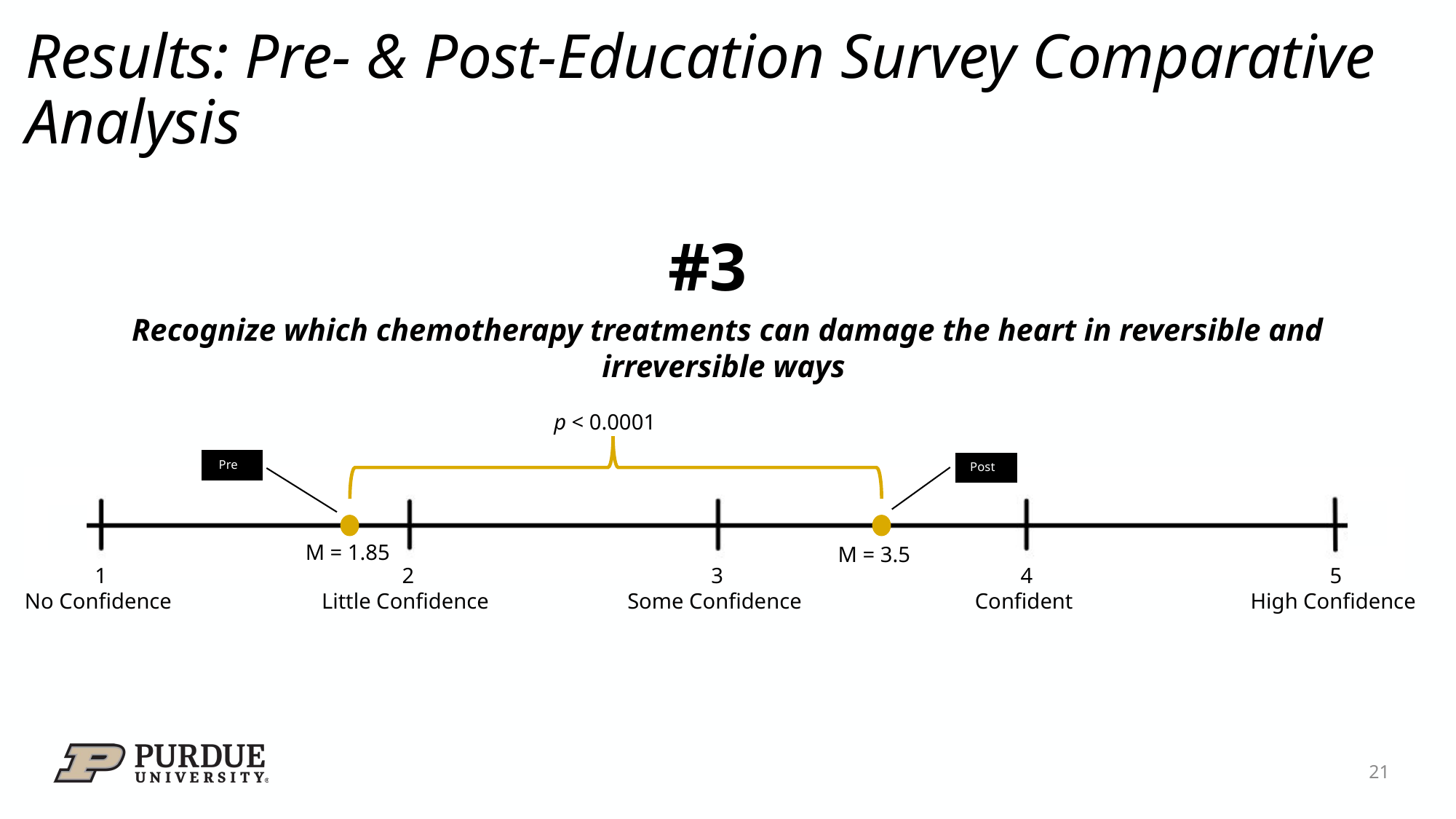

# Results: Pre- & Post-Education Survey Comparative Analysis
#3
Recognize which chemotherapy treatments can damage the heart in reversible and irreversible ways
p < 0.0001
Pre
Post
1
No Confidence
2
Little Confidence
3
Some Confidence
4
Confident
5
High Confidence
M = 1.85
M = 3.5
21

## Slide 22
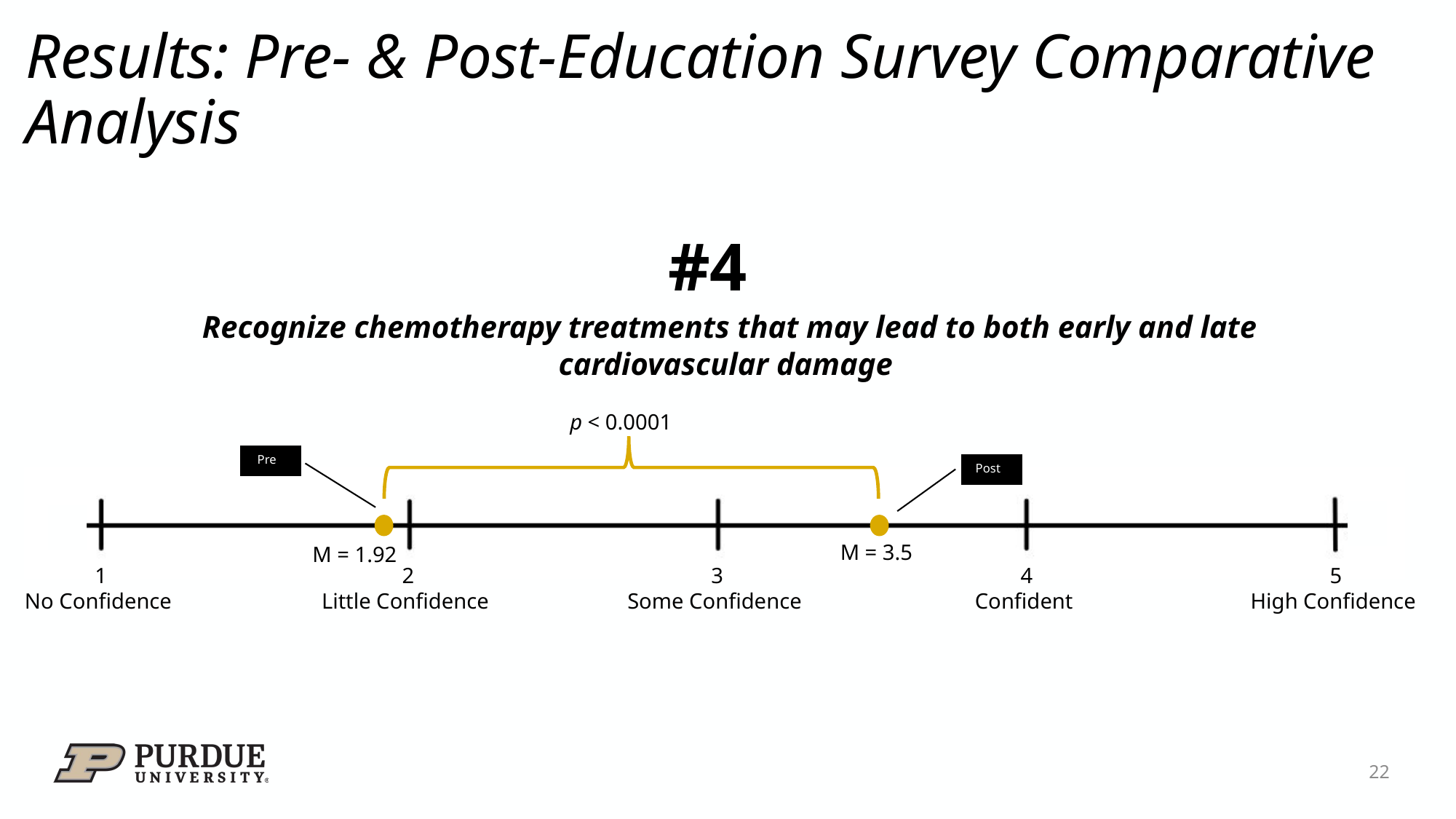

# Results: Pre- & Post-Education Survey Comparative Analysis
#4
Recognize chemotherapy treatments that may lead to both early and late cardiovascular damage
p < 0.0001
Pre
Post
1
No Confidence
2
Little Confidence
3
Some Confidence
4
Confident
5
High Confidence
M = 3.5
M = 1.92
22

## Slide 23
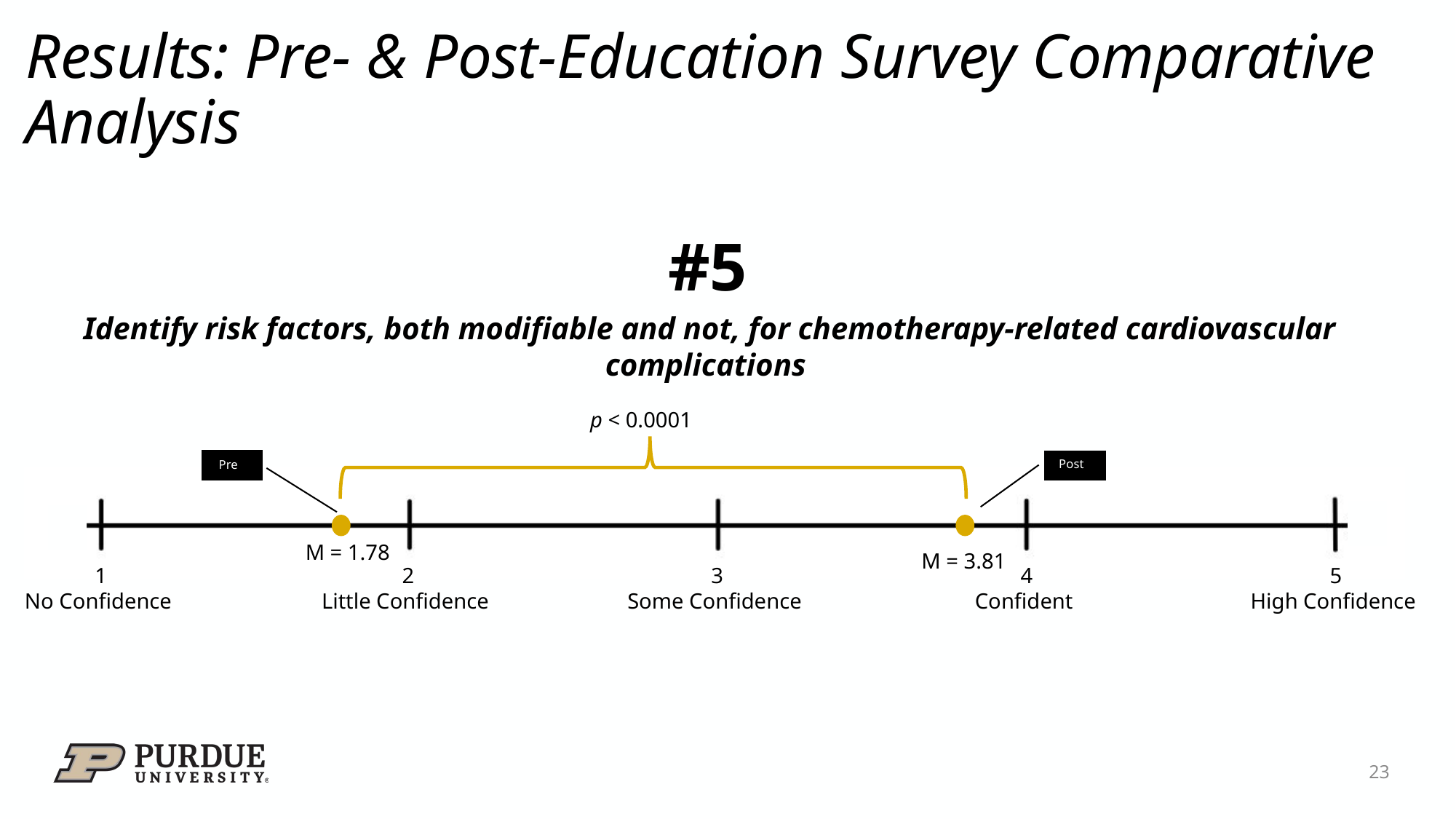

# Results: Pre- & Post-Education Survey Comparative Analysis
#5
Identify risk factors, both modifiable and not, for chemotherapy-related cardiovascular complications
p < 0.0001
Pre
Post
1
No Confidence
2
Little Confidence
3
Some Confidence
4
Confident
5
High Confidence
M = 1.78
M = 3.81
23

## Slide 24
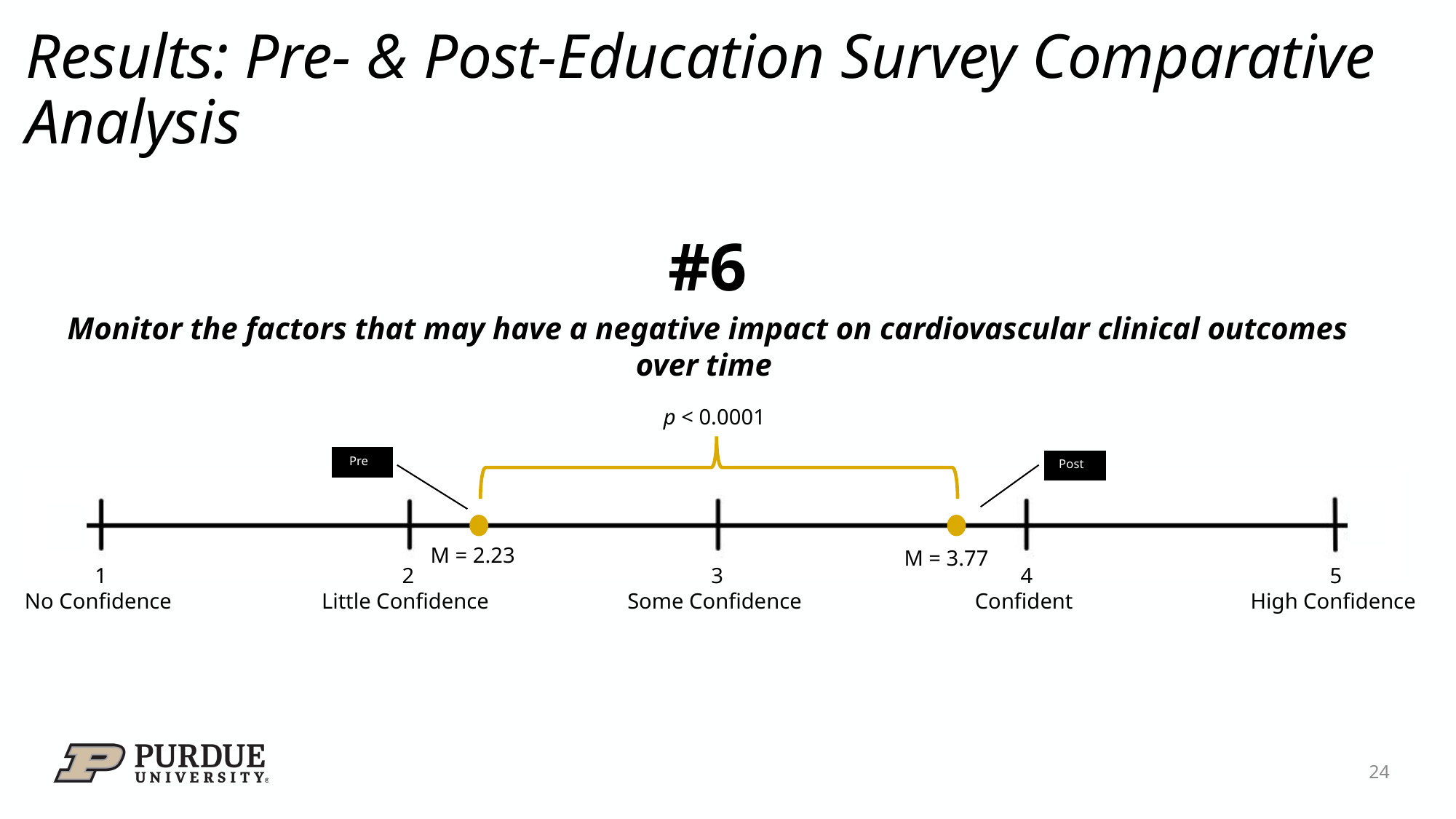

# Results: Pre- & Post-Education Survey Comparative Analysis
#6
Monitor the factors that may have a negative impact on cardiovascular clinical outcomes over time
p < 0.0001
Pre
Post
1
No Confidence
2
Little Confidence
3
Some Confidence
4
Confident
5
High Confidence
M = 2.23
M = 3.77
24

## Slide 25
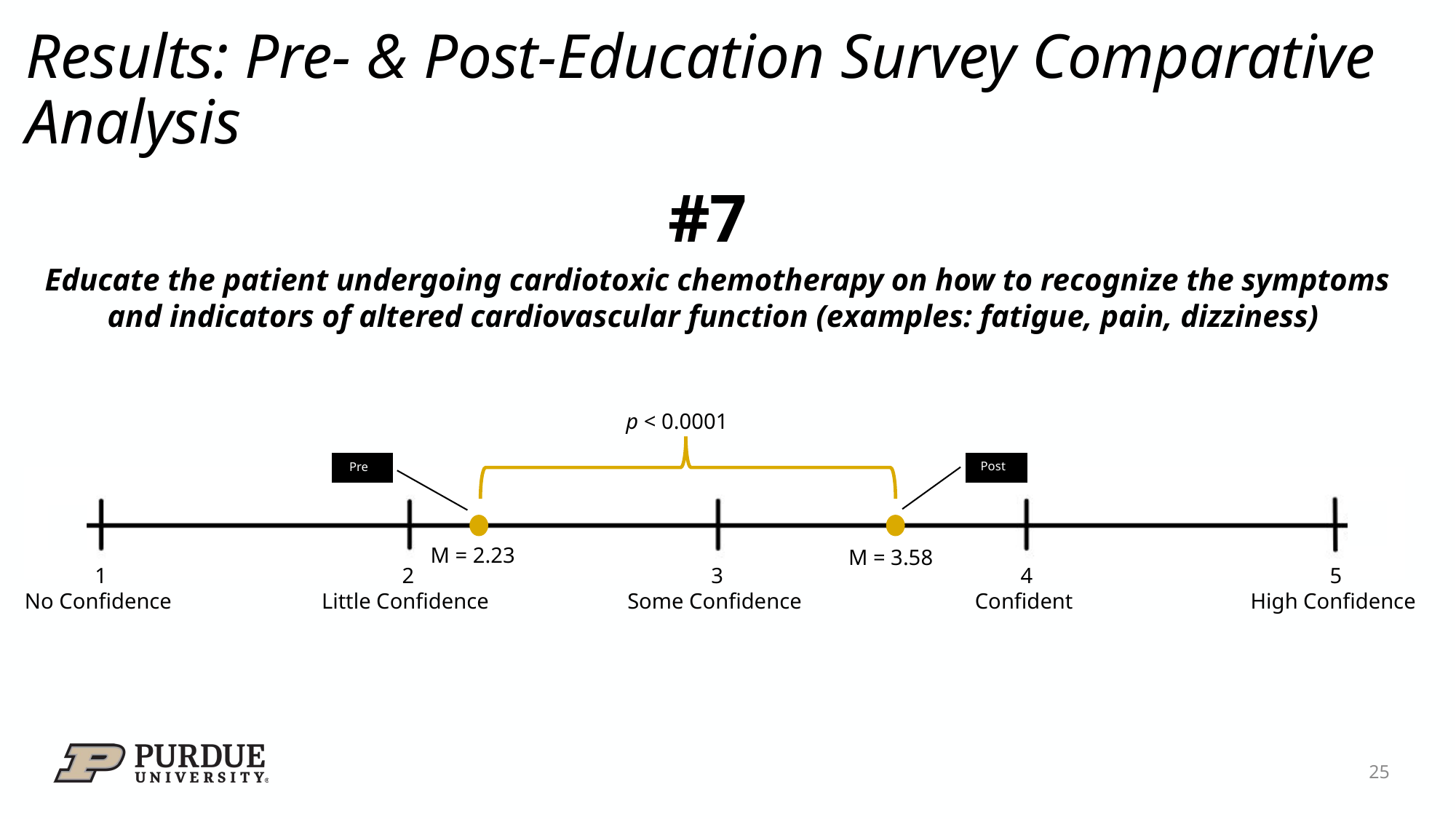

# Results: Pre- & Post-Education Survey Comparative Analysis
#7
Educate the patient undergoing cardiotoxic chemotherapy on how to recognize the symptoms and indicators of altered cardiovascular function (examples: fatigue, pain, dizziness)
p < 0.0001
Pre
Post
1
No Confidence
2
Little Confidence
3
Some Confidence
4
Confident
5
High Confidence
M = 2.23
M = 3.58
25

## Slide 26
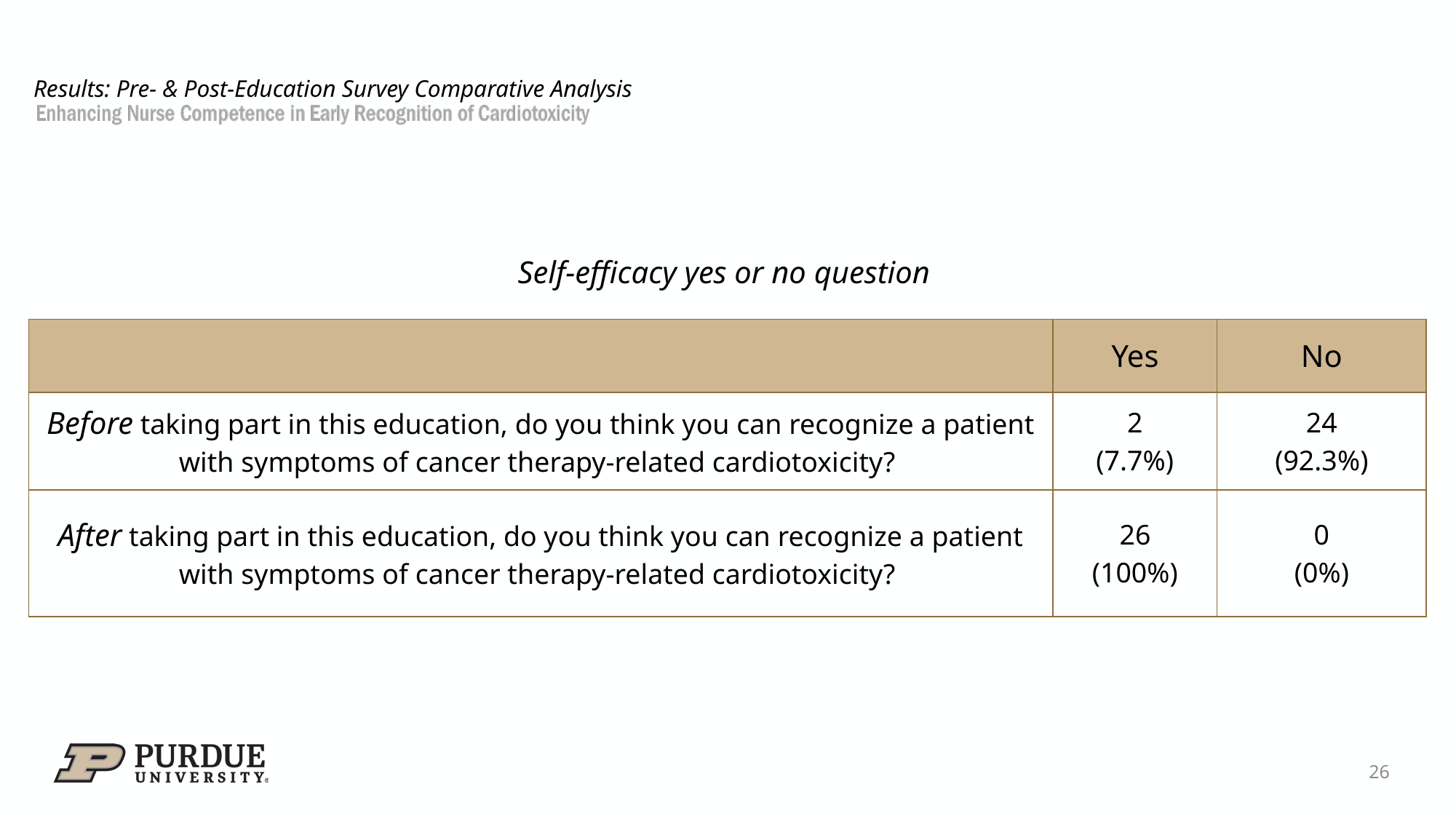

# Results: Pre- & Post-Education Survey Comparative Analysis
Self-efficacy yes or no question
| | Yes | No |
| --- | --- | --- |
| Before taking part in this education, do you think you can recognize a patient with symptoms of cancer therapy-related cardiotoxicity? | 2 (7.7%) | 24 (92.3%) |
| After taking part in this education, do you think you can recognize a patient with symptoms of cancer therapy-related cardiotoxicity? | 26 (100%) | 0 (0%) |
26

## Slide 27
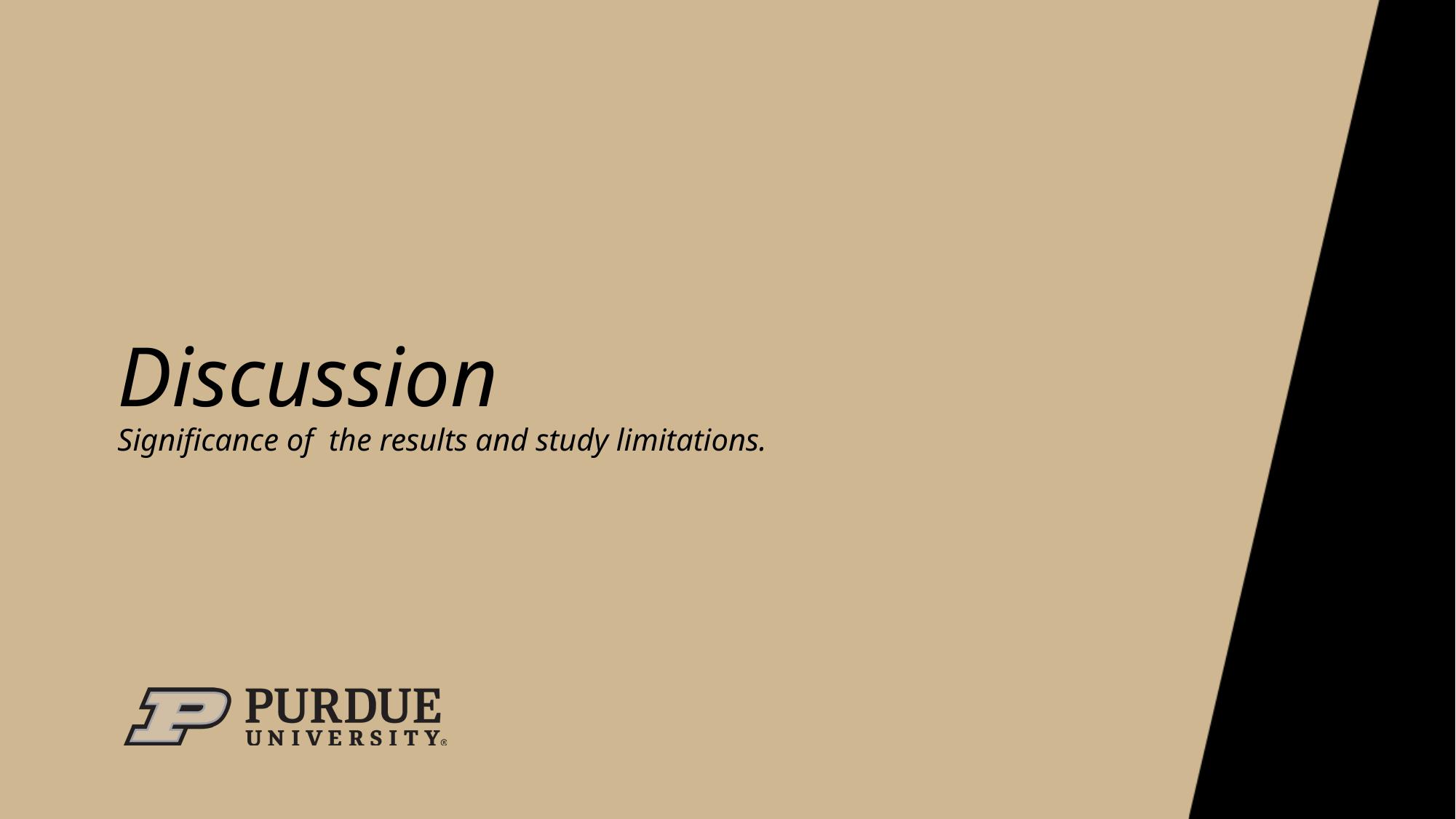

# Discussion Significance of the results and study limitations.

## Slide 28
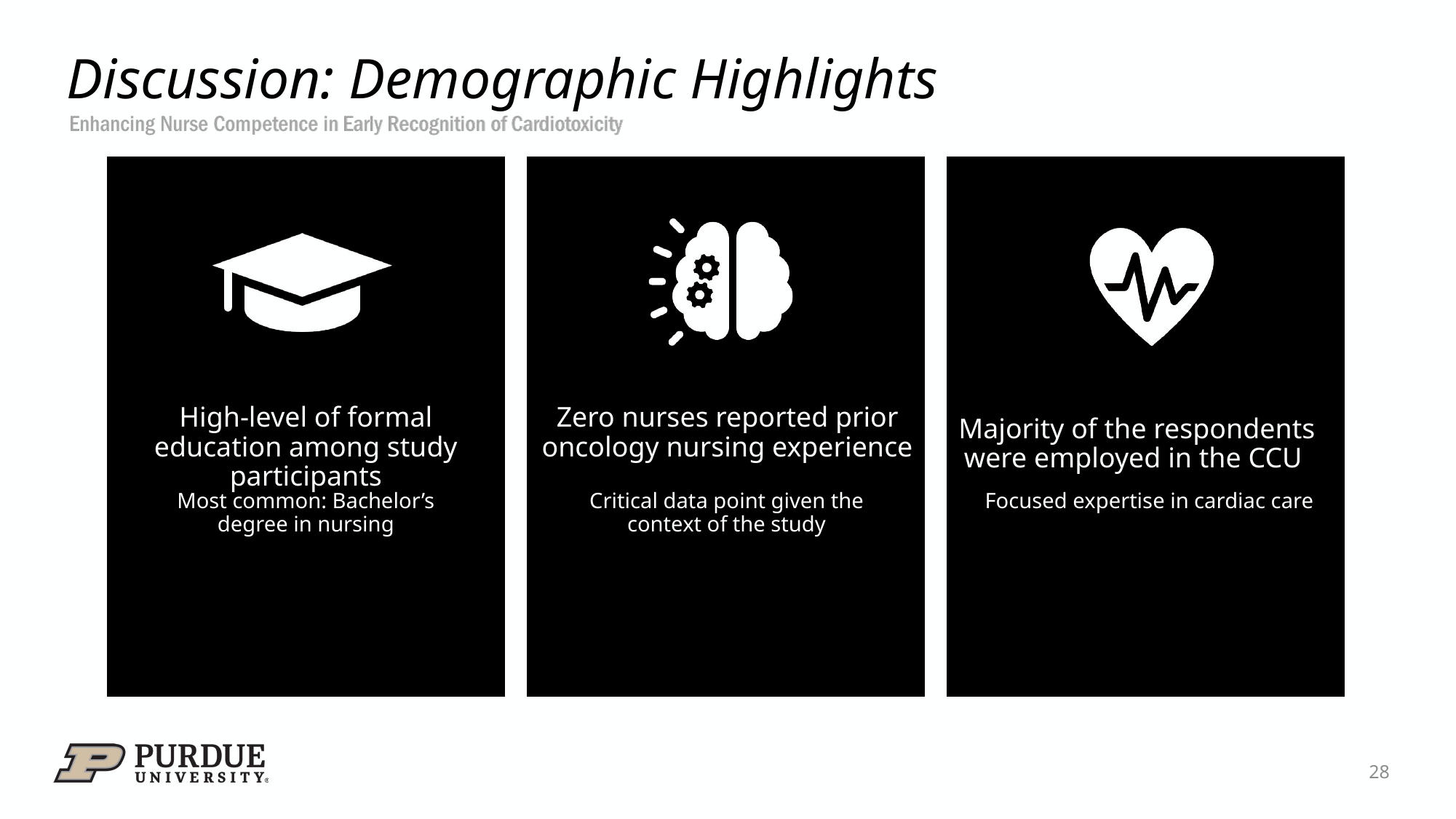

# Discussion: Demographic Highlights
High-level of formal education among study participants
Zero nurses reported prior oncology nursing experience
Majority of the respondents were employed in the CCU
Most common: Bachelor’s degree in nursing
Critical data point given the context of the study
Focused expertise in cardiac care care
28

## Slide 29
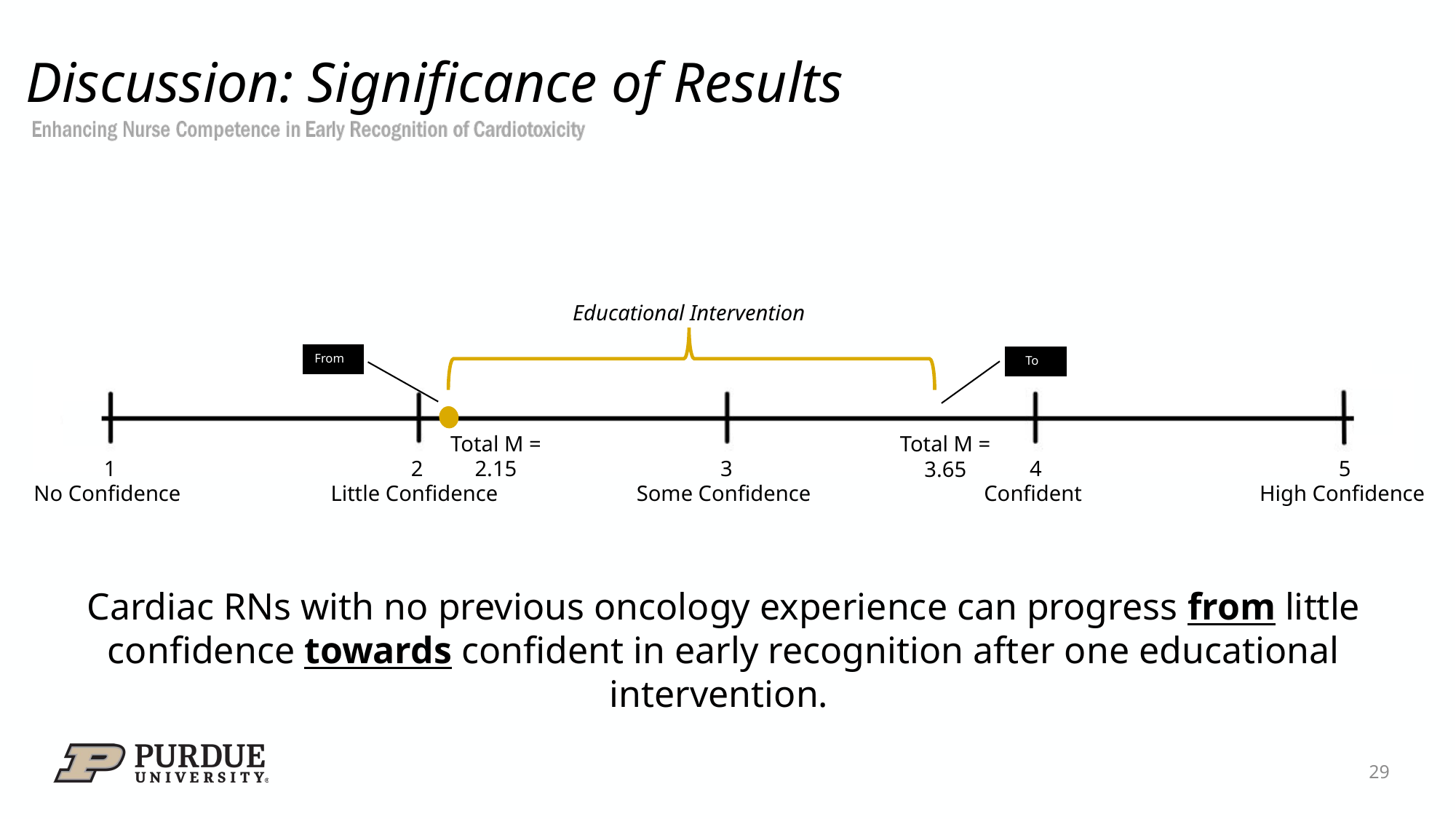

# Discussion: Significance of Results
Cardiac RNs with no previous oncology experience can progress from little confidence towards confident in early recognition after one educational intervention.
Educational Intervention
From
To
1
No Confidence
2
Little Confidence
3
Some Confidence
4
Confident
5
High Confidence
Total M = 2.15
Total M = 3.65
29

## Slide 30
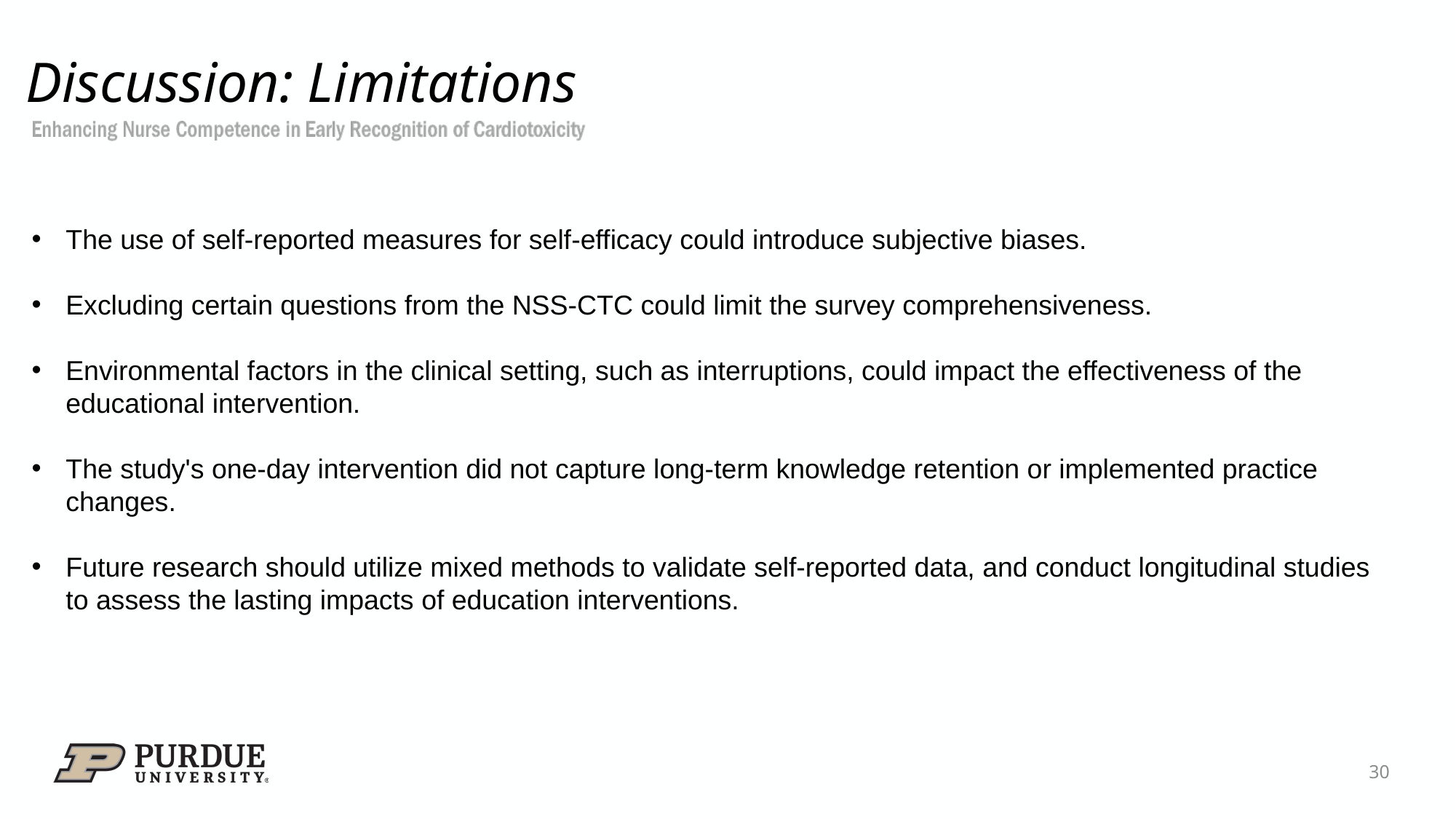

# Discussion: Limitations
The use of self-reported measures for self-efficacy could introduce subjective biases.
Excluding certain questions from the NSS-CTC could limit the survey comprehensiveness.
Environmental factors in the clinical setting, such as interruptions, could impact the effectiveness of the educational intervention.
The study's one-day intervention did not capture long-term knowledge retention or implemented practice changes.
Future research should utilize mixed methods to validate self-reported data, and conduct longitudinal studies to assess the lasting impacts of education interventions.
30

## Slide 31
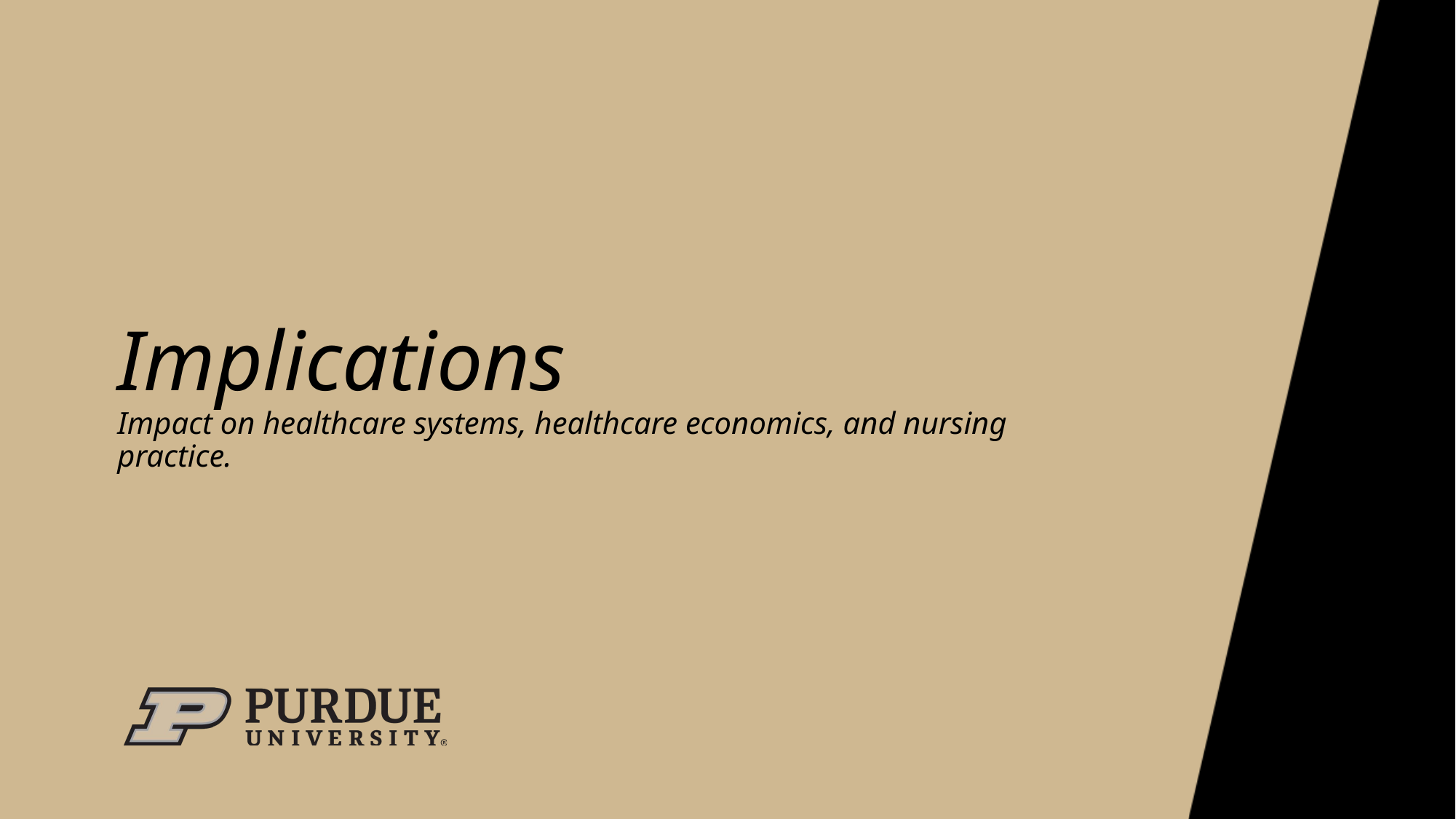

# Implications Impact on healthcare systems, healthcare economics, and nursing practice.

## Slide 32
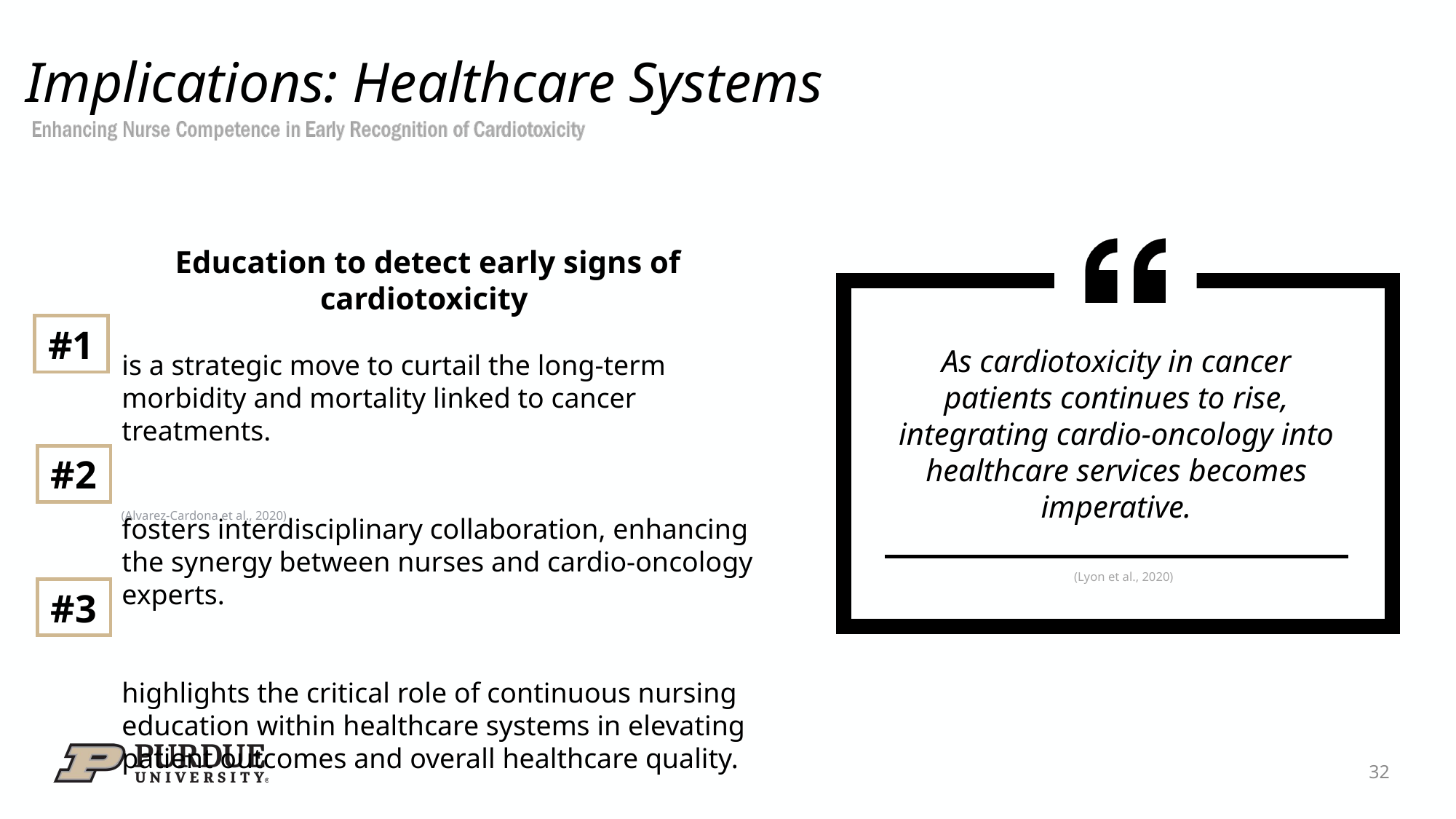

# Implications: Healthcare Systems
As cardiotoxicity in cancer patients continues to rise, integrating cardio-oncology into healthcare services becomes imperative.
(Lyon et al., 2020)
Education to detect early signs of cardiotoxicity
is a strategic move to curtail the long-term morbidity and mortality linked to cancer treatments.
fosters interdisciplinary collaboration, enhancing the synergy between nurses and cardio-oncology experts.
highlights the critical role of continuous nursing education within healthcare systems in elevating patient outcomes and overall healthcare quality.
#1
#2
(Alvarez-Cardona et al., 2020)
#3
32

## Slide 33
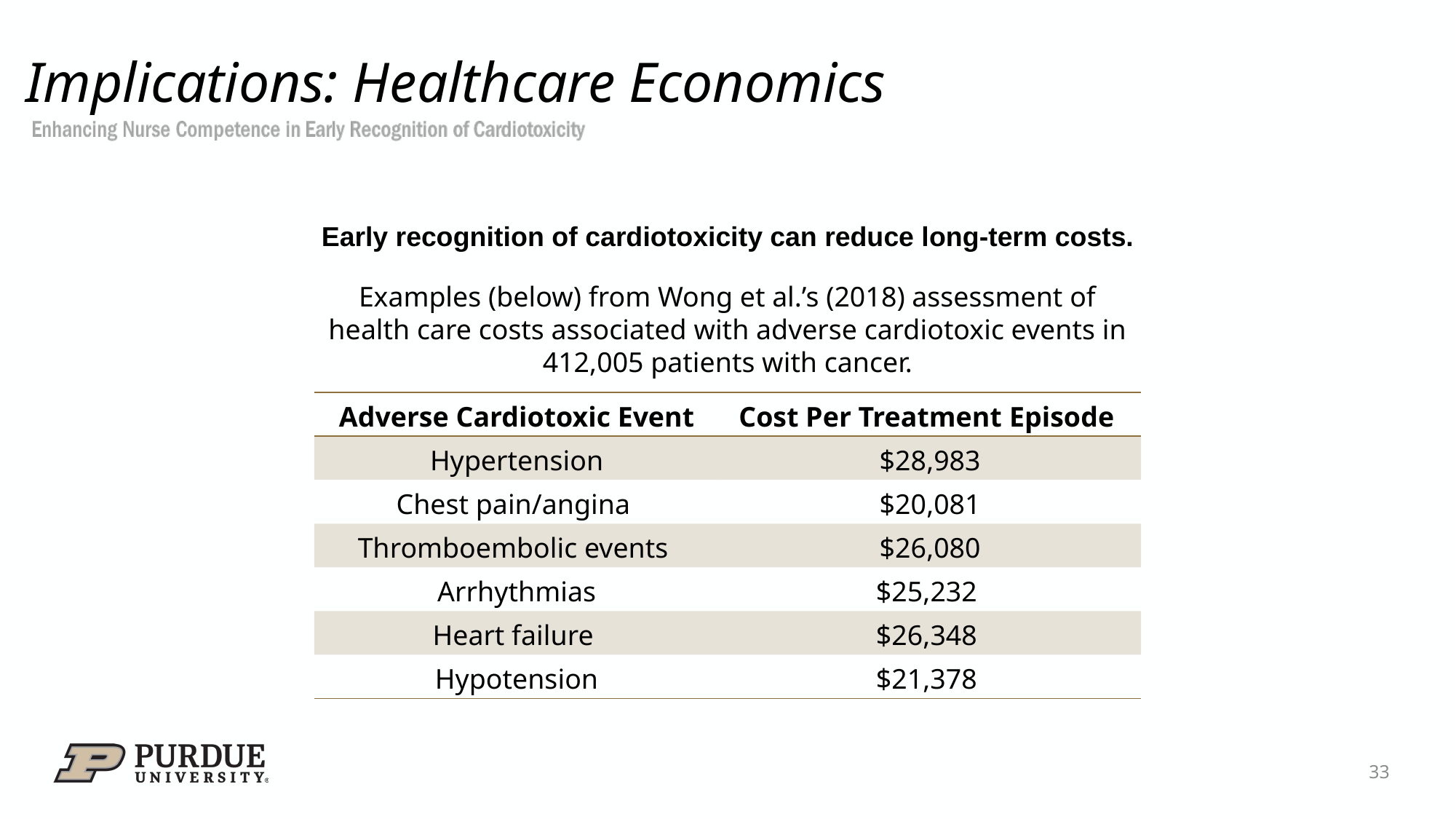

# Implications: Healthcare Economics
Early recognition of cardiotoxicity can reduce long-term costs.
Examples (below) from Wong et al.’s (2018) assessment of health care costs associated with adverse cardiotoxic events in 412,005 patients with cancer.
| Adverse Cardiotoxic Event | Cost Per Treatment Episode |
| --- | --- |
| Hypertension | $28,983 |
| Chest pain/angina | $20,081 |
| Thromboembolic events | $26,080 |
| Arrhythmias | $25,232 |
| Heart failure | $26,348 |
| Hypotension | $21,378 |
33

## Slide 34
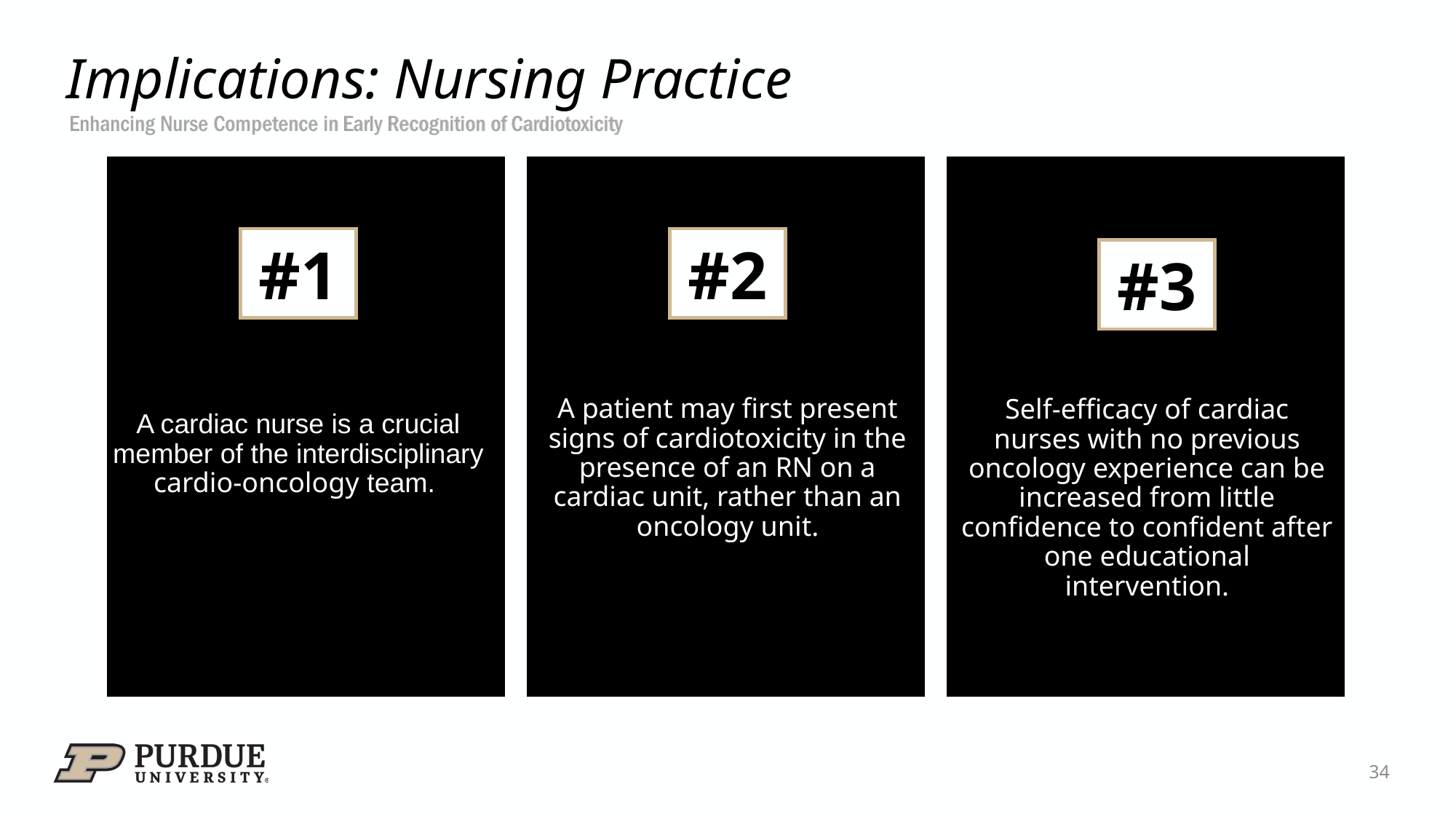

# Implications: Nursing Practice
#1
#2
#3
Self-efficacy of cardiac nurses with no previous oncology experience can be increased from little confidence to confident after one educational intervention.
A cardiac nurse is a crucial member of the interdisciplinary cardio-oncology team.
A patient may first present signs of cardiotoxicity in the presence of an RN on a cardiac unit, rather than an oncology unit.
34

## Slide 35
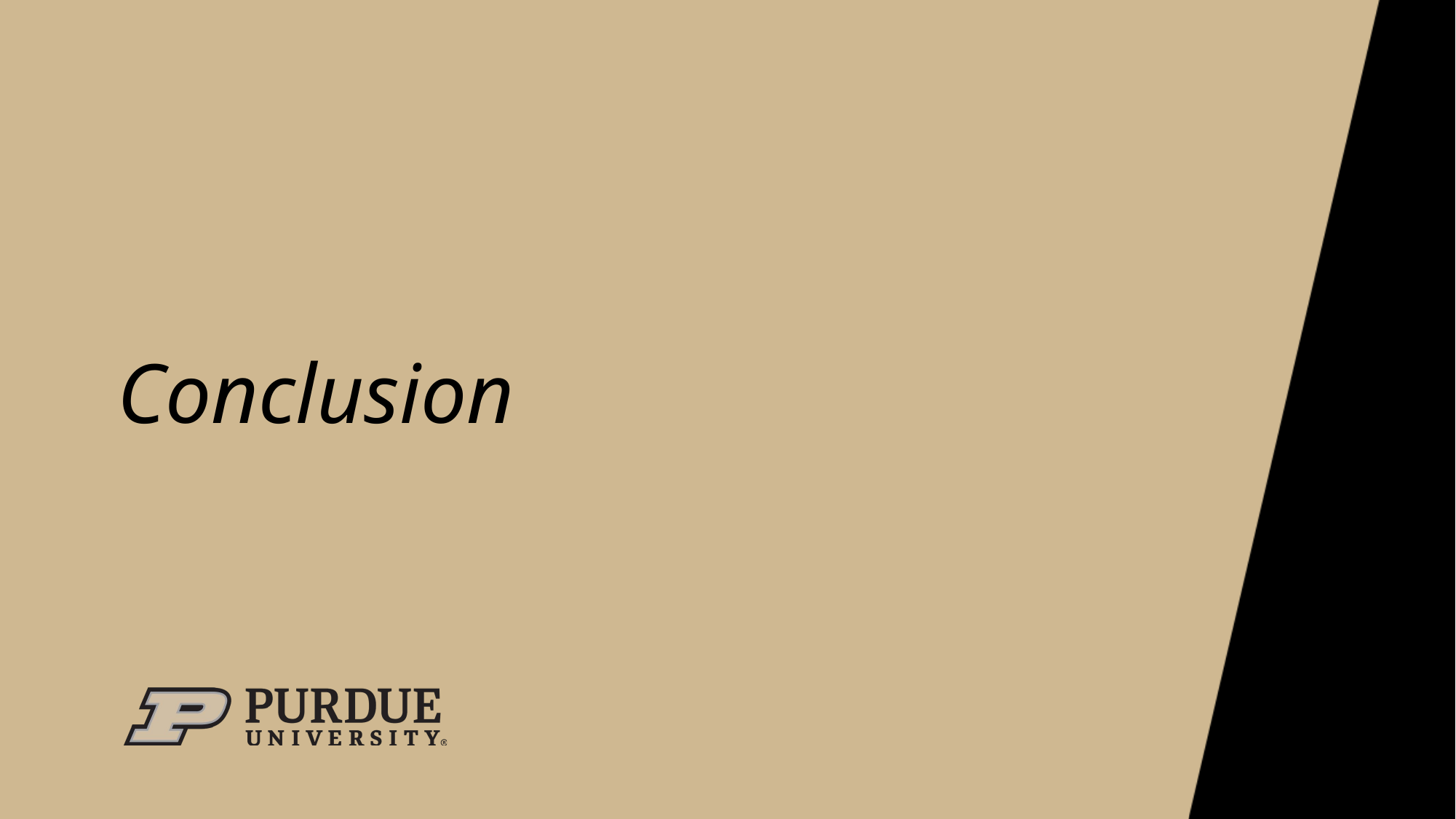

# Conclusion

## Slide 36
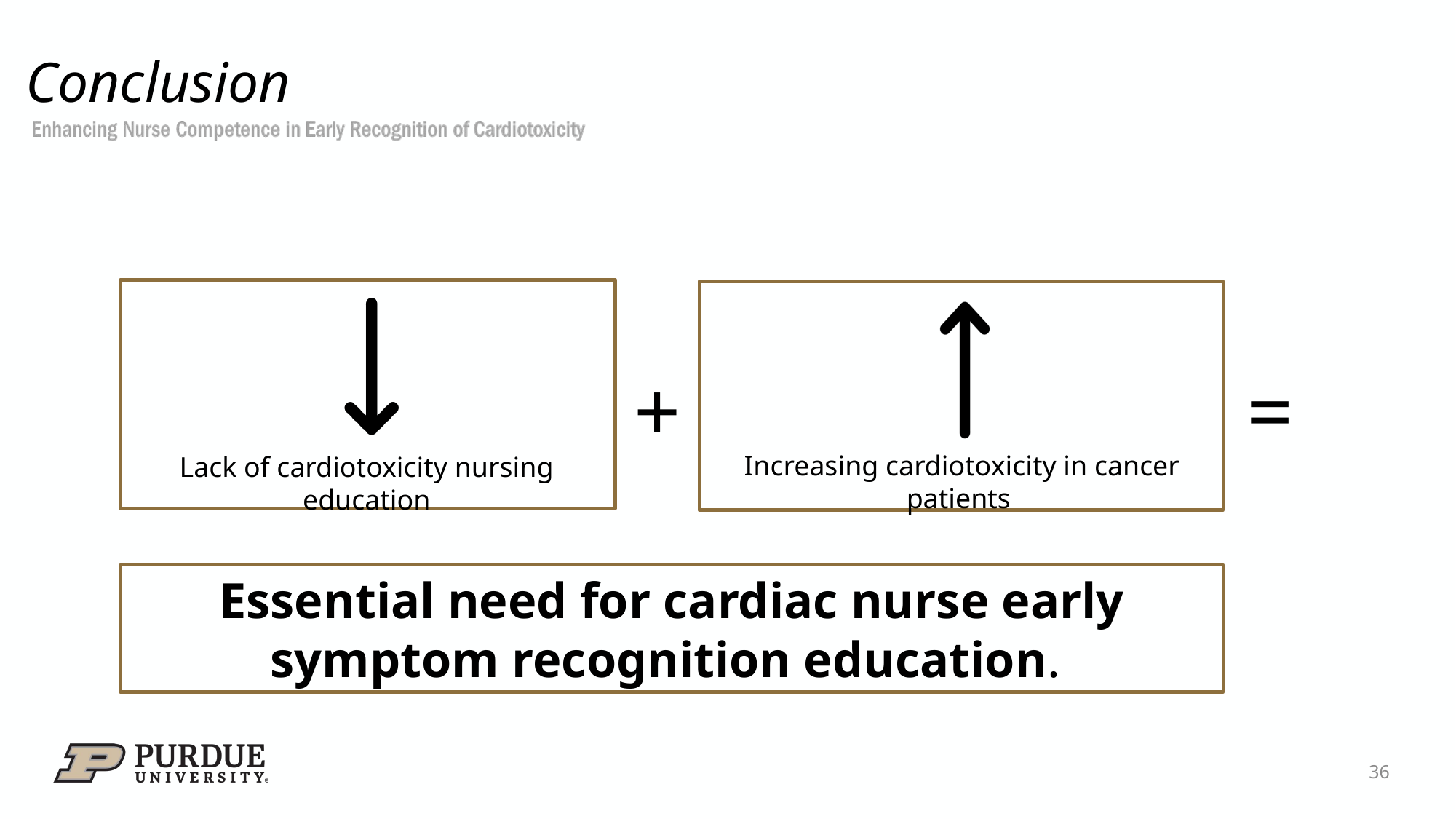

# Conclusion
Lack of cardiotoxicity nursing education
Increasing cardiotoxicity in cancer patients
+
=
Essential need for cardiac nurse early symptom recognition education.
36

## Slide 37
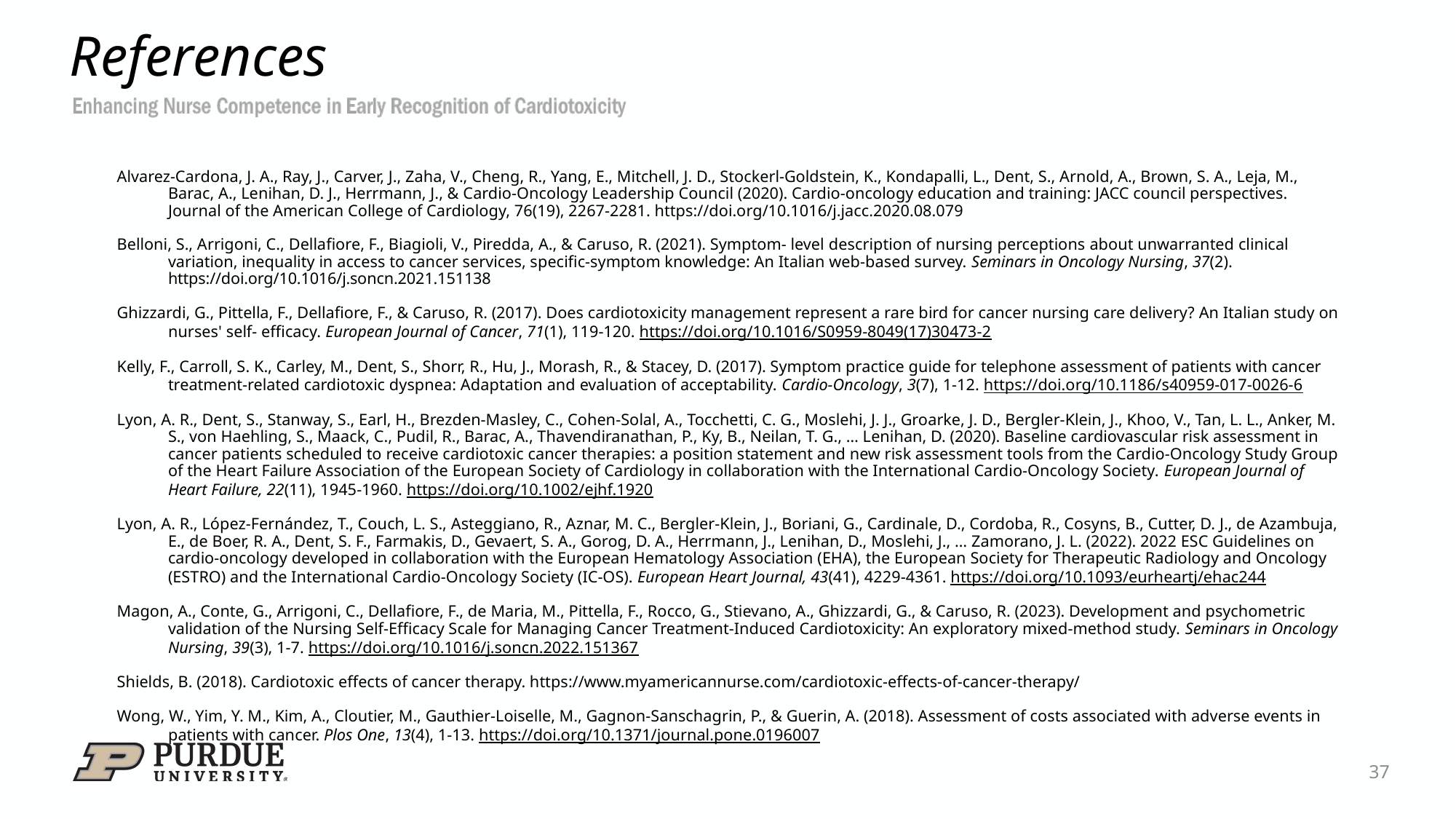

# References
Alvarez-Cardona, J. A., Ray, J., Carver, J., Zaha, V., Cheng, R., Yang, E., Mitchell, J. D., Stockerl-Goldstein, K., Kondapalli, L., Dent, S., Arnold, A., Brown, S. A., Leja, M., Barac, A., Lenihan, D. J., Herrmann, J., & Cardio-Oncology Leadership Council (2020). Cardio-oncology education and training: JACC council perspectives. Journal of the American College of Cardiology, 76(19), 2267-2281. https://doi.org/10.1016/j.jacc.2020.08.079
Belloni, S., Arrigoni, C., Dellafiore, F., Biagioli, V., Piredda, A., & Caruso, R. (2021). Symptom- level description of nursing perceptions about unwarranted clinical variation, inequality in access to cancer services, specific-symptom knowledge: An Italian web-based survey. Seminars in Oncology Nursing, 37(2). https://doi.org/10.1016/j.soncn.2021.151138
Ghizzardi, G., Pittella, F., Dellafiore, F., & Caruso, R. (2017). Does cardiotoxicity management represent a rare bird for cancer nursing care delivery? An Italian study on nurses' self- efficacy. European Journal of Cancer, 71(1), 119-120. https://doi.org/10.1016/S0959-8049(17)30473-2
Kelly, F., Carroll, S. K., Carley, M., Dent, S., Shorr, R., Hu, J., Morash, R., & Stacey, D. (2017). Symptom practice guide for telephone assessment of patients with cancer treatment-related cardiotoxic dyspnea: Adaptation and evaluation of acceptability. Cardio-Oncology, 3(7), 1-12. https://doi.org/10.1186/s40959-017-0026-6
Lyon, A. R., Dent, S., Stanway, S., Earl, H., Brezden-Masley, C., Cohen-Solal, A., Tocchetti, C. G., Moslehi, J. J., Groarke, J. D., Bergler-Klein, J., Khoo, V., Tan, L. L., Anker, M. S., von Haehling, S., Maack, C., Pudil, R., Barac, A., Thavendiranathan, P., Ky, B., Neilan, T. G., … Lenihan, D. (2020). Baseline cardiovascular risk assessment in cancer patients scheduled to receive cardiotoxic cancer therapies: a position statement and new risk assessment tools from the Cardio-Oncology Study Group of the Heart Failure Association of the European Society of Cardiology in collaboration with the International Cardio-Oncology Society. European Journal of Heart Failure, 22(11), 1945-1960. https://doi.org/10.1002/ejhf.1920
Lyon, A. R., López-Fernández, T., Couch, L. S., Asteggiano, R., Aznar, M. C., Bergler-Klein, J., Boriani, G., Cardinale, D., Cordoba, R., Cosyns, B., Cutter, D. J., de Azambuja, E., de Boer, R. A., Dent, S. F., Farmakis, D., Gevaert, S. A., Gorog, D. A., Herrmann, J., Lenihan, D., Moslehi, J., … Zamorano, J. L. (2022). 2022 ESC Guidelines on cardio-oncology developed in collaboration with the European Hematology Association (EHA), the European Society for Therapeutic Radiology and Oncology (ESTRO) and the International Cardio-Oncology Society (IC-OS). European Heart Journal, 43(41), 4229-4361. https://doi.org/10.1093/eurheartj/ehac244
Magon, A., Conte, G., Arrigoni, C., Dellafiore, F., de Maria, M., Pittella, F., Rocco, G., Stievano, A., Ghizzardi, G., & Caruso, R. (2023). Development and psychometric validation of the Nursing Self-Efficacy Scale for Managing Cancer Treatment-Induced Cardiotoxicity: An exploratory mixed-method study. Seminars in Oncology Nursing, 39(3), 1-7. https://doi.org/10.1016/j.soncn.2022.151367
Shields, B. (2018). Cardiotoxic effects of cancer therapy. https://www.myamericannurse.com/cardiotoxic-effects-of-cancer-therapy/
Wong, W., Yim, Y. M., Kim, A., Cloutier, M., Gauthier-Loiselle, M., Gagnon-Sanschagrin, P., & Guerin, A. (2018). Assessment of costs associated with adverse events in patients with cancer. Plos One, 13(4), 1-13. https://doi.org/10.1371/journal.pone.0196007
37
