# Supplementary material for: Enhancing nurse competence in early recognition of cardiotoxicity
Source: Cardiooncology. 2024 Sep 14;10:62. doi: 10.1186/s40959-024-00261-x (PMC11401397; doi:10.1186/s40959-024-00261-x)
Supplement: Supplementary file 2 — Supplementary Material 2 [file 40959_2024_261_MOESM2_ESM.docx]

# Appendix A

*Pre-Project Survey*

**Section 1: Demographic Questions**

**Age:**

20-30

31-40

41-50

51+

**Gender Identity:**

Female Male Transgender Other

Prefer not to answer

**Employment Status**

Full-time Part-time PRN

**Years practice as a registered nurse**

Less than 1 year 1-4 years

5-9 years

10-14 years

15-19 years

Greater than 20 years

**What is the highest nursing degree you have completed?**

Associate Degree in Nursing Bachelor’s Degree in Nursing Master’s Degree in Nursing

Doctorate Degree in Nursing (PhD, ScD, DNS, ND, DNP

**What is the highest non-nursing degree you have completed?**

Associate Degree (Non-nursing) Bachelor’s Degree (Non-nursing) Master’s Degree (Non-nursing)

Doctorate in Non-Nursing field (PhD, JD, MD,EdD) None

**Do you have previous oncology nursing experience?**

Yes No

**What is your primary unit of employment?**

CCU

Resource Travel Float

**Section 2: Confidence & Recognition Check**

Please select the level of confidence for each of the following statements.

**Before taking part in this education do you think you can recognize a patient with symptoms of cancer therapy-related cardiotoxicity?**

Yes No

**Monitor for signs and symptoms of deteriorating cardiovascular functions over time**

No confidence Little confidence Some confidence Confidence

High confidence

**Recognize the signs and symptoms of secondary cardiotoxicity caused by chemotherapy treatments**

No confidence Little confidence Some confidence Confidence

High confidence

**Recognize which chemotherapy treatments can damage the heart in reversible and irreversible ways**

No confidence Little confidence Some confidence Confidence

High confidence

**Recognize chemotherapy treatments that may lead to both early and late cardiovascular damage**

No confidence Little confidence Some confidence Confidence

High confidence

**Identify risk factors, both modifiable and not, for chemotherapy-related cardiovascular complications**

No confidence Little confidence Some confidence Confidence

High confidence

**Monitor the factors that may have a negative impact on cardiovascular clinical outcomes over time**

No confidence Little confidence Some confidence Confidence

High confidence

**Educate the patient undergoing cardiotoxic chemotherapy on how to recognize the symptoms and indicators of altered cardiovascular function (examples: fatigue, pain, dizziness)**

No confidence Little confidence Some confidence Confidence

High confidence

# Appendix B

*Post-Project Survey*

**Confidence & Recognition Check**

Please select the level of confidence for each of the following statements.

**After taking part in this education do you think you can recognize a patient with symptoms of cancer therapy-related cardiotoxicity?**

Yes No

**Monitor for signs and symptoms of deteriorating cardiovascular functions over time**

No confidence Little confidence Some confidence Confidence

High confidence

**Recognize the signs and symptoms of secondary cardiotoxicity caused by chemotherapy treatments**

No confidence Little confidence Some confidence Confidence

High confidence

**Recognize which chemotherapy treatments can damage the heart in reversible and irreversible ways**

No confidence Little confidence Some confidence Confidence

High confidence

**Recognize chemotherapy treatments that may lead to both early and late cardiovascular damage**

No confidence Little confidence Some confidence Confidence

High confidence

**Identify risk factors, both modifiable and not, for chemotherapy-related cardiovascular complications**

No confidence Little confidence Some confidence Confidence

High confidence

**Monitor the factors that may have a negative impact on cardiovascular clinical outcomes over time**

No confidence Little confidence Some confidence Confidence

High confidence

**Educate the patient undergoing cardiotoxic chemotherapy on how to recognize the symptoms and indicators of altered cardiovascular function (examples: fatigue, pain, dizziness)**

No confidence Little confidence Some confidence Confidence

High confidence

# Appendix C

*CardioToxicity Management Self-Efficacy Scale (NSS-CTC)*

**Please, considering a typical working day, I can…**

| Completely no confidence | Quite Confidence | Moderately Confidence | Very Confidence | Completely Confidence |
| --- | --- | --- | --- | --- |
| **1** | **2** | **3** | **4** | **5** |

| **1.** Recognize which chemotherapy treatments can damage the heart in  reversible and irreversible ways | **1** | **2** | **3** | **4** | **5** |
| --- | --- | --- | --- | --- | --- |
| **2.** Recognize chemotherapy treatments that may lead to both early and  late cardiovascular damage | **1** | **2** | **3** | **4** | **5** |
| **3.** Recognize the symptoms and signs of secondary cardiotoxicity caused by chemotherapy treatments | **1** | **2** | **3** | **4** | **5** |
| **4.** Identify risk factors, both modifiable and not, for chemotherapy- related cardiovascular complications | **1** | **2** | **3** | **4** | **5** |
| **5**. Adjust the modifiable risk factors resulting from the use of cardiotoxic chemotherapeutic treatments | **1** | **2** | **3** | **4** | **5** |
| **6**. Find and interpret the recommendations and evidence available in the literature related to chemotherapy cardiotoxicity | **1** | **2** | **3** | **4** | **5** |
| **7.** Utilize the recommendations and evidence on chemotherapy cardiotoxicity that are available in the literature | **1** | **2** | **3** | **4** | **5** |
| **8**. Interpret the results of laboratory tests to determine their predictive  value of cardiovascular damage | **1** | **2** | **3** | **4** | **5** |
| **9.** Determine the levels of understanding, knowledge, and lifestyles (for example, eating habits, physical activity, smoking, and sedentary behaviors) in a patient receiving cardiotoxic chemotherapeutic treatments | **1** | **2** | **3** | **4** | **5** |
| **10**. Monitor for signs and symptoms of deteriorating cardiovascular functions over time | **1** | **2** | **3** | **4** | **5** |
| **11.** Monitor the factors that may have a negative impact on cardiovascular clinical outcomes over time | **1** | **2** | **3** | **4** | **5** |
| **12.** Monitor lifestyle changes over time and compliance with  cardiotoxicity recommendations | **1** | **2** | **3** | **4** | **5** |
| **13.** Provide cardiotoxic chemotherapy patients with information about healthy lifestyles to adopt (example: nutrition, physical  activity, smoking cessation) | **1** | **2** | **3** | **4** | **5** |
| **14.** Educate the patient undergoing cardiotoxic chemotherapy on how to recognize the symptoms and indicators of altered  cardiovascular function (examples: fatigue, pain, dizziness) | **1** | **2** | **3** | **4** | **5** |
| 1**5.** Educate the patient undergoing cardiotoxic chemotherapy on how to control the variables that may have a detrimental impact on  their clinical cardiovascular outcomes (examples: chronic diseases  such as arterial hypertension and diabetes mellitus) | **1** | **2** | **3** | **4** | **5** |
